# Characterization of the genomic landscape of canine oral osteosarcoma reveals similarities with appendicular osteosarcoma

**DOI:** 10.1371/journal.pone.0325181

**Published:** 2025-06-10

**Authors:** Christopher Husted, Sarah Adrianowycz, Cornelia Peterson, Suzanne Bartholf DeWitt, Elinor K. Karlsson, William Eward, Jason A. Somarelli, Kate Megquier, Heather L. Gardner

**Affiliations:** 1 UMass Chan Medical School, Worcester, Massachusetts, United States of America; 2 Broad Institute of MIT and Harvard, Cambridge, Massachusetts, United States of America; 3 Tufts University Cummings School of Veterinary Medicine, North Grafton, Massachusetts, United States of America; 4 Department of Medicine, Duke University Medical Center, Durham, North Carolina, United States of America; 5 Department of Orthopaedic Surgery, Duke University Medical Center, Durham, North Carolina, United States of America; Universita degli Studi della Campania Luigi Vanvitelli, ITALY

## Abstract

Osteosarcoma (OS) is the most common bone tumor in both dogs and humans. It predominantly occurs in the appendicular skeleton, with about 25% of cases occurring within the axial skeleton. Progression of local disease is often the life-limiting factor for patients with axial OS, in contrast to appendicular OS, where local disease is addressed surgically, and metastatic disease remains the primary obstacle. While OS is a rare human cancer, limiting the availability of samples, its higher incidence in dogs provides a valuable comparative model for study. Both canines and humans share commonalities in clinical presentation, but dogs have an accelerated progression. Similarly, complex structural genetic changes define appendicular OS in both dogs and people, but it is unclear whether the genomic landscape of axial OS exhibits different alterations that may separate it from appendicular OS. We performed pilot whole genome sequencing of canine primary oral (maxillary or mandibular) OS tumors (n = 8) and matched normal tissue. We found that the genetics of canine oral OS largely parallel the genetics of canine appendicular OS, including an overall low number of recurrent point mutations affecting the same gene (*TP53* and *SETD2,* 3/8 samples). Structural variants dominated the landscape of mutational changes, with recurrent variants in *DMD* (4/8) and *DLG2* (3/8) found at a similar incidence to appendicular OS. This pilot suggests genomic similarities between oral and appendicular OS.

## Introduction

Osteosarcoma (OS) is the most common primary bone tumor in pediatric patients. While most patients develop OS in the appendicular skeleton, which includes bones of the limbs, primary tumors affecting the axial skeleton, including the spine, skull, and ribcage have also been reported. OS is uncommon and affects fewer than 1,000 people in the United States annually [1], and axial OS accounts for approximately 11% of this rare disease in people aged 0–24 [2]. Due to the proximity of axial tumors to critical structures, local tumor control and subsequent metastatic disease remain a pervasive clinical challenge. For people with unresectable axial tumors, 3-year survival rates are low, ranging from 11–56% [3]. Improved treatment options for patients with unresectable tumors or metastatic disease are needed, and a comprehensive knowledge of the tumor genome is important for enabling therapeutic development and precision medicine [4,5].

Canine OS is a valuable model system for osteosarcoma due to the approximately 10-fold higher disease incidence (estimated 13.9 compared to 1.02 per 100,000 dog or person-years at risk respectively), similar clinical presentation, and development of chemotherapy-resistant pulmonary metastatic disease [1,6]. Dogs also enable the evaluation of novel therapeutics in the setting of treatment-naive tumors and minimal residual disease [7]. As in people, appendicular OS is the most common form of OS in dogs, with approximately 12% of dogs developing the axial form of the disease [8]. The genomic landscape of canine appendicular OS is similar to the human disease, with copy number alterations in *MYC* and *DLG2* and a predominance of complex structural alterations [9]. Recent genomic characterization has clarified the unique advantages of this naturally occurring large animal model, underscoring its potential to bridge gaps between human patients and other model systems and enhance relevant human studies [1,7].

Oral OS, affecting the mandible or maxilla, is the most common form of axial OS [2,10,11]. It is unknown whether oral OS is genetically distinct from the more common appendicular OS. This information is critical to inform whether outcome-linked genetic characterization in appendicular OS is applicable to oral OS. The genomic landscape of human oral osteosarcoma remains largely unexplored, with whole-genome sequencing (WGS) data from only four samples of jaw or mandibular osteosarcoma published [12–14]. These limited datasets suggest that the genetic landscapes of oral and appendicular OS are similar; however, the small amount of available data precludes definitive conclusions.

There are no published WGS datasets of canine oral OS. Therefore, we conducted a pilot study of eight oral OS tumor-normal pairs with the main objectives of (1) exploring the genetic landscape of canine oral OS, and (2) performing a preliminary comparison of canine oral OS to canine appendicular OS.

## Materials and methods

### Sample acquisition and DNA extraction

Matched canine primary tumor and normal tissue samples were provided by The Ohio State University Biospecimen Repository (n = 7), and the Canine Comparative Oncology Consortium (CCOGC) [15] (n = 1). Genomic DNA was isolated from flash-frozen tumors and normal tissue samples using the DNeasy Blood & Tissue Kit (Qiagen Inc., Hilden, Germany) per manufacturer instructions. Sample collection was approved by the Institutional Animal Care and Use Committees at the collecting institutions (The Ohio State University protocol number 2010A0015-R5, University of Wisconsin-Madison protocol number V005297).

### Histopathological assessment

Hematoxylin and eosin-stained (H&E) tissue sections were available for review by a single board-certified veterinary pathologist for 5 of the 8 samples to confirm the initial clinical diagnosis and histologic subtype and to estimate the percentage of tumor and necrosis in each sample. Tumor fraction was computationally estimated in all eight samples using the ichorCNA tool [16], as histologic slides were unavailable for review in 3 cases [16].

### Library construction and sequencing

Library preparation and whole genome sequencing (WGS) were performed by the Broad Institute Genomics Platform, as previously described [9]. Briefly, 100 nanograms of genomic DNA was prepared using the KAPA Hyper Prep Kit with Library Amplification Primer Mix (KAPA Biosystems; #KK8504) with palindromic forked adaptors containing a unique 8-base index sequence (Roche). Libraries (2.2nM each) were pooled and sequenced on an Illumina HiSeqX using 151 base pair paired-end reads. Normal tissue samples were sequenced to a target depth of 30x and tumors to a target depth of 60x. The mean sequencing depth achieved was 51x (range 43X - 57x) for normal samples and 95x (range 70x - 119x) for tumor samples.

### Preprocessing of sequencing data

Fastq files were aligned to the canine reference genome (CanFam4, UU_Cfam_GSD_1.0 [17] with the Y chromosome sequence from the ROS_Cfam_1.0 assembly appended [18,19]) using BWA, and were preprocessed following GATK best practices [17–23]. For all GATK tools, version 4.2.3.0 was used unless otherwise stated. Duplicate reads were identified using Picard Tools MarkDuplicates (http://broadinstitute.github.io/picard). Base Quality Score Recalibration (BQSR) was performed using a VCF file of germline variants from 1987 dog and other canid samples [18,23].

### Simple somatic mutation calling

**Fig 1:**
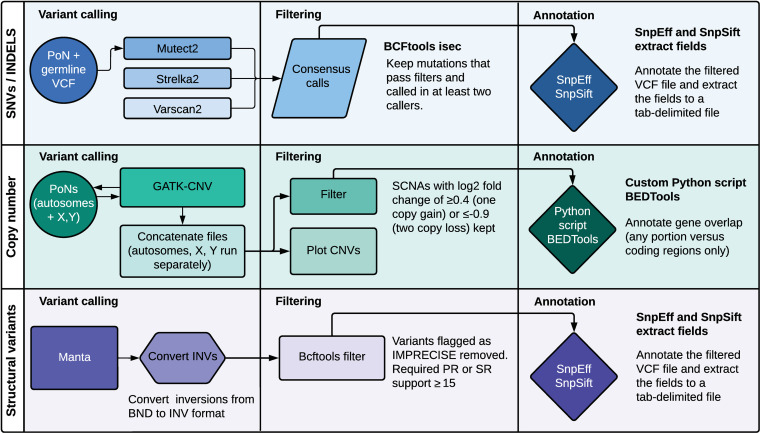
Overview of somatic calling workflow. Workflow showing the tools and settings used to call simple somatic variants, copy number variants, and other structural variants.

Simple somatic mutations (single nucleotide variants (SNVs) and small insertions and deletions (indels)) were detected using a consensus calling approach combining three somatic mutation callers: Mutect2, Strelka2, and VarScan2 (**Fig 1**) [24–26]. Mutect2 was run with the germline reference file from 1987 dogs and other canids and a panel of normals of 40 dogs (including the eight reported in this study). Additional arguments “—downsampling-stride 20,” “—max-reads-per-alignment-start 6,” and “—max-suspicious-reads-per-alignment-start 6” were used in running Mutect2. FilterMutectCalls was run with the “—run_orientation_bias_mixture_model_filter” option set to “True” and the “—min-median-read-position” option set to 10 bp. A VCF file containing common (allele frequency between 0.01 and 0.2) germline variants was used in the CalculateContamination step. The default settings for tumor-normal calling were used for Strelka2 and VarScan2. The mutation calls from each tool that passed filtering were processed through bcftools [27] isec to obtain a consensus. Only mutation calls identified in two or more callers were retained for downstream analysis. The KaryoploteR package, using R (R4.4.0), was used to identify areas of kataegis [28]. Lollipop plots were created using the lollipops tool [29].

### Structural variant calling

Structural variants (SVs) were called using Manta version 1.6.0 in the tumor-normal configuration [30]. The output VCFs were processed using the Manta-provided script “convertInversion.py” to convert inversions to the older INV format rather than the break end (BND) format. The calls were then processed using bcftools, removing any calls where the “FILTER” flag was not set to “PASS”, that were marked as imprecise (IMPRECISE = 1), or where neither the paired read (PR) or split read (SR) support was 15 reads or more.

### Somatic copy number aberration calling

Somatic copy number aberrations (SCNAs) were detected using the GATK somatic CNV pipeline in tumor-normal mode [20,21]. Four panels of normals were created using germline samples from dogs included in this study, plus 32 additional dogs with a cancer diagnosis—one containing the autosomes, male-only and female-only panels for chromosome X, and one male-only panel for chromosome Y. SCNAs were called on the autosomes and chromosomes X and Y separately, as recommended. Copy number losses with a log2 fold change of ≥0.4 (one copy gain) or ≤−0.9 (two copy loss) were considered for analysis.

### Oncoprint visualization

The filtered somatic mutations were plotted as an oncoprint using the PyOncoprint [25] Python package, with additional annotations added in Adobe Illustrator 2023. Genes were included in the oncoprint based on the following criteria: (1) genes with alterations in more than three samples; (2) genes with alterations in three samples and either commonly reported in the OS literature or found in the COSMIC Cancer Gene Census Tier 1 genes; (3) genes that were recurrently altered in our dataset and not previously reported in canine OS; and (4) genes that were recurrently altered in our dataset and linked to inherited osteosarcoma syndromes, bone cancer, or sarcoma in the COSMIC database [12,31,32].

Structural variants are displayed as “multi-hits” when more than two variant types are present within the same gene in a single sample. Simple somatic mutations are represented as “multi-effect” when a single mutation results in multiple effects in one or more gene transcripts.

### Coding exon overlap

To summarize somatic copy number aberrations and other structural variants which overlap coding regions of genes, we used the BEDTools [33] “annotate” function to count the number of mutations in each sample overlapping the CDS regions of the CanFam4 genome. These counts were then aggregated to the gene and sample levels.

### Mutational signature calling

The SigProfilerMatrixGenerator tool was used to generate a matrix of variant mutational contexts, and all samples were run through CrossMap (v0.7.0) to lift over the mutations from CanFam4 to Canfam3.1 [34,35]. We then used the SigFit tool (v2.2) to identify the COSMIC v3 single base substitution (SBS) signatures in the oral osteosarcoma data [12,36]. Fitting was run with 10000 iterations and 5000 warmup iterations using the multinomial model. Signatures sufficiently greater than zero (meaning that the lower end of the Bayesian HPD interval was > 0.025 in any sample) were selected, and fitting was rerun using only those signatures.

### Pathway analyses

Pathway analysis was performed using the STRING Database [37] app on the Cytoscape desktop tool, version 3.10.3 [38]. Species was set to *Canis lupus familiaris*, background was set to “whole genome”, confidence score cutoff was set at 0.5, and maximum additional interactors were set at 0. Gene lists for individual pathways were downloaded from MSigDB [39,40], including “Chromatin Modifying Enzymes” and “Neurexins and neuroligins” from Reactome [41], “Cell Cycle” and “MAPK Signaling Pathway” from the Kyoto Encyclopedia of Genes and Genomes (KEGG) [42], and “Phosphatidylinositol 3 Kinase Protein Kinase B Signal Transduction” from the Gene Ontology Biological Processes [43,44]. Protein interactions were defined using the STRING Database and The Human Protein Atlas [45,46].

## Results

### Cohort characteristics

Patient demographics were consistent with published data [47,48]. The median age at diagnosis was 9 years (range 5–12 years). The most common breeds were Labrador retriever (n = 3) and mixed breed (n = 3). The other included breeds (n = 1 each) were boxer and bullmastiff. Five of eight (62.5%) dogs were female (four spayed, one intact), and three were castrated males. Two tumors were maxillary in origin, and six were mandibular in origin. Full patient demographic and clinical metadata is available in S1 Table.

### Histopathological assessment

**Fig 2:**
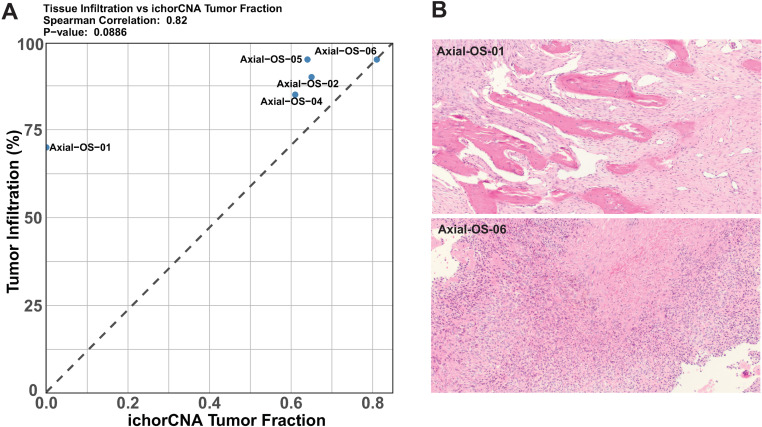
Assessment of tumor content. A. Correlation plot of the samples where we have both tumor fraction obtained from ichorCNA and percent neoplastic cells in the tumor mass as determined by a pathologist. B. Histological images of two canine oral osteosarcoma samples with the highest and lowest fraction of tumor infiltration.

H&E slides were available for 5 of the 8 samples for review by a pathologist to confirm the diagnosis of OS, histologic subtype, and estimate the percentage of tumor tissue and necrosis in each sample (Fig 2, S1 Fig). Four tumors were mandibular and one maxillary. Five OS subtypes were represented: chondroblastic, fibroblastic, mixed, osteoblastic, and poorly differentiated osteoblastic. The approximate percentage of the proportion of neoplastic cells ranged from 70–95%, with a median of 87%. The estimated percentage of tissue necrosis ranged widely from <10% to 40%, with a median of approximately 15%. Since H&E slides were not available for review in 3 dogs (Axial-OS-03, Axial-OS-07, Axial-OS-08), tumor content was also assessed computationally using the ichorCNA tool. The histologic and computational assessment of tumor content were highly correlated (Spearman’s rho = 0.82, p-value: 0.09) (**Fig 2A**). The ichorCNA derived tumor fractions ranged from 0–81%, with a median of 64% (**Fig 2B**). One sample (Axial-OS-01) was an outlier, with the pathologist estimate of neoplastic cells of 70%, but an ichorCNA tumor fraction estimate of 0. The cause of this discrepancy is unknown, but possible contributing factors include sampling disparities between the frozen and fixed specimens in the context of tumor heterogeneity and low numbers of large-scale copy number changes in the tumor.

### Simple somatic mutations in canine oral OS

**Fig 3:**
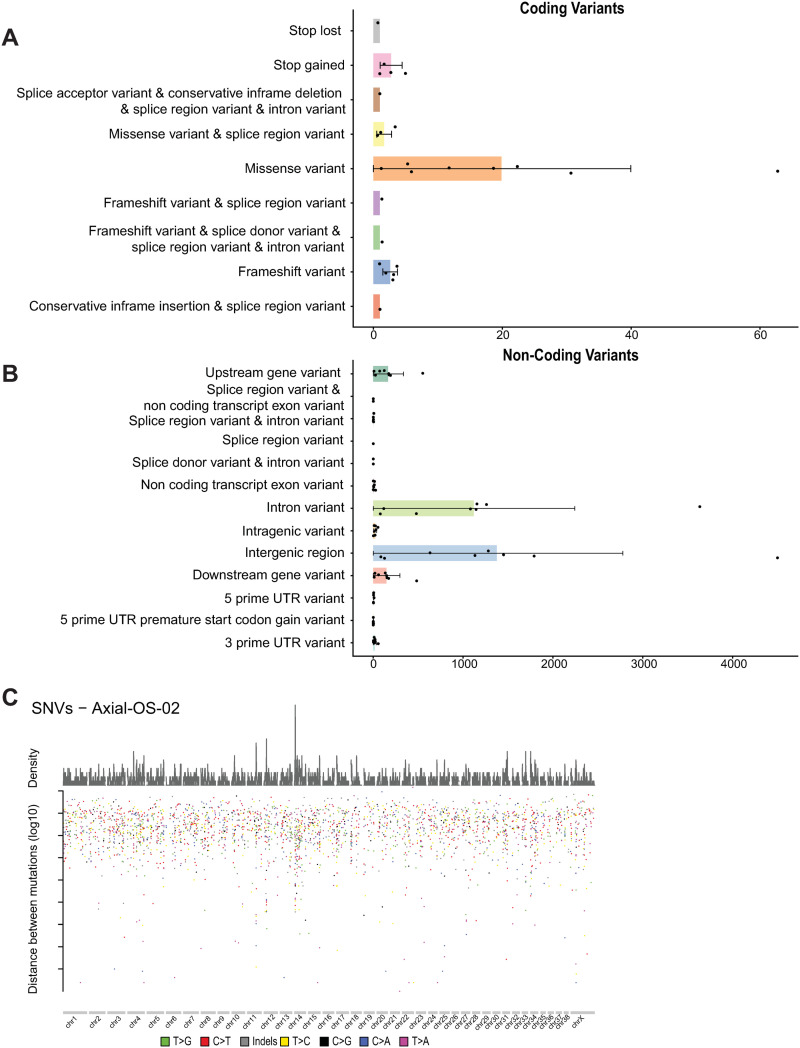
A. Frequency of coding single nucleotide variants and B. non-coding single nucleotide variants; C. Rainfall plot showing focal hypermutation in Axial-OS-02.

**Fig 4:**
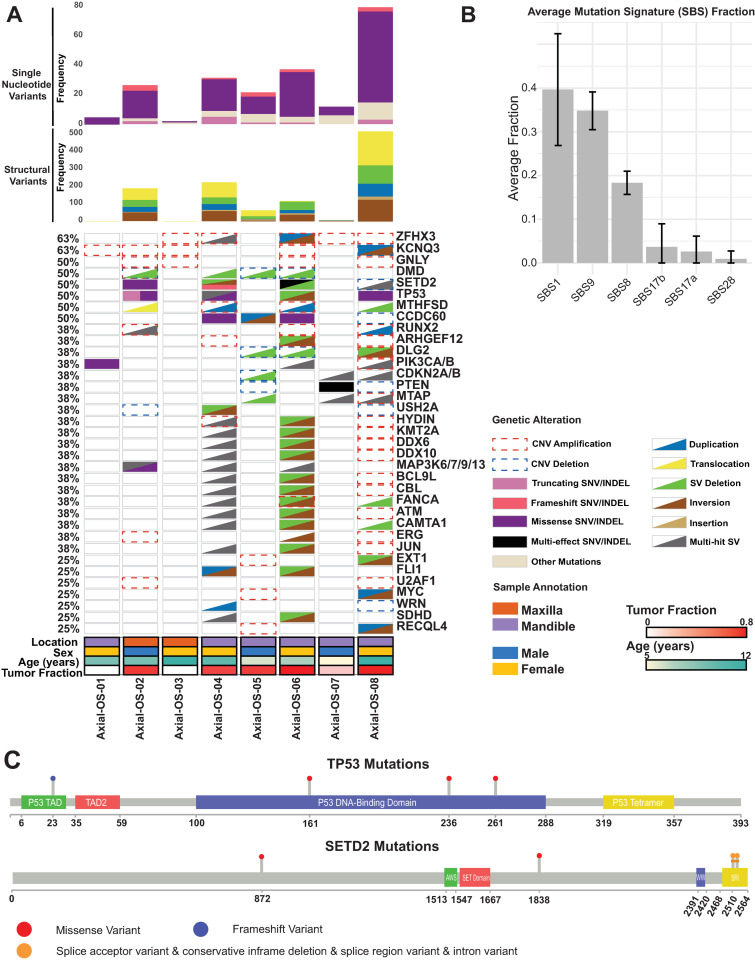
A. Oncoprint represents the key simple somatic mutations, copy number aberrations, and other structural variants in recurrently altered or known osteosarcoma genes; B. Barplots summarize the most common single base substitution (SBS) signatures. Error bars represent the mean exposure ± standard deviation (SD); C. Lollipop plots showing mutations in TP53 and SETD2 proteins.

We identified recurrent mutations in key genes such as *TP53* (n = 3) and *SETD2* (n = 3). The median number of simple somatic mutations (SNVs or INDELs) per sample was 21 coding (range 1–72) and 2702 non-coding (range 188–9327). The non-coding and coding SNV counts per sample were highly correlated (r = 0.95, p = 0.0002). Missense mutations were the most common coding SNV identified (64%) (Fig 3). Consistent with findings in canine appendicular OS samples [9,49,50], *TP53* and *SETD2* were mutated in multiple samples (3/8; 38%) (Fig 4, S3 Table). In *TP53*, we observed several mutations across multiple dogs in the P53 DNA binding domain and one frameshift mutation in the P53 transactivation domain. *SETD2* mutations were not clustered in a particular domain or location (Fig 4). Five additional genes had recurrent coding mutations (2/8 samples each, 25%) –– *DNAI2* and *SH3BGRL2*, which have been shown to be differentially expressed in human OS [51,52], and *CCDC60*, *FCRL5*, and *IMMP2L*, which have not previously been reported in OS.

Noncoding SNVs and INDELs (within untranslated regions (UTRs), introns, or upstream or downstream of the gene) were identified in several genes mutated in published human and dog OS datasets. Four genes had noncoding variants associated with them in all 8 samples, all of which have been previously described as altered in human or canine OS: *DMD*, *DLG2* [9,53], *CCSER1* [54], and *NAALADL2* [55]. An additional nine genes were associated with noncoding mutations in 7/8 (88%) of samples, including *MAGI2*, *CTNND2,* and *CSMD1*, which have been reported to be altered in OS [53].

In canine appendicular OS, regions of focal hypermutations indicative of kataegis have been reported [9]. Using a modified maftools [56] “kataegis_detect_chr” function, we identified a single hypermutated region on chromosome 14 in the Axial-OS-02 sample (Fig 3). No distinct patterns of hypermutation were detected in the other samples (S2 Fig).

### Structural variants in canine oral OS

**Fig 5:**
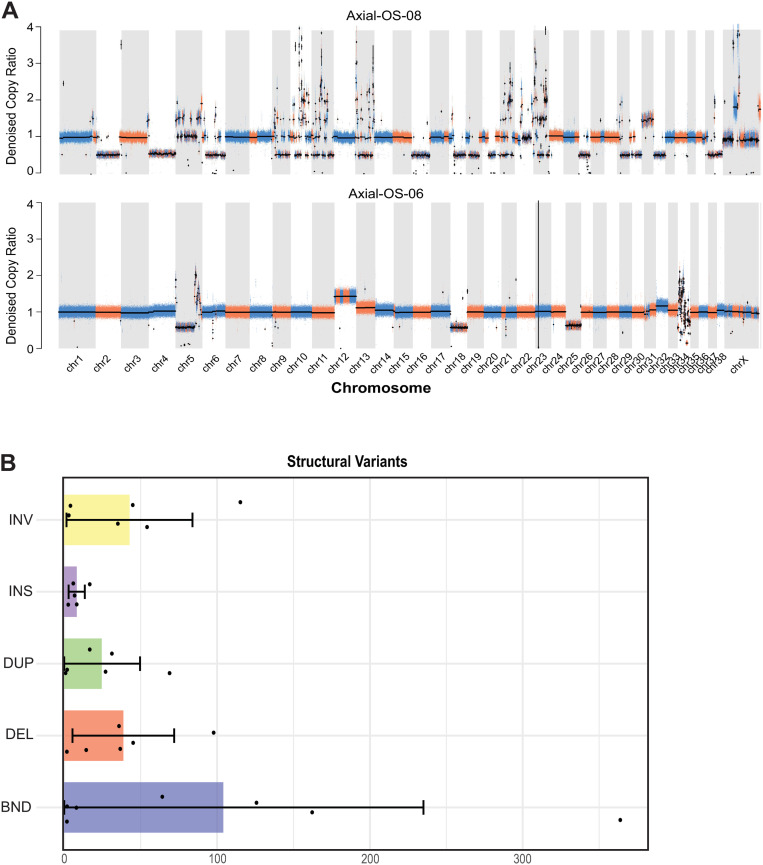
A. Copy Number ratio plots illustrating the locations of both focal and whole-chromosome copy number aberrations in Axial-OS-08 and Axial-OS-06; B. Barplots of the frequency of Structural Variant types across canine oral osteosarcoma samples. Error bars represent the mean exposure ± standard deviation (SD), with the lower bound adjusted to zero if below this value.

Structural variants (SVs) were identified, including translocations, inversions, insertions, deletions, and duplications (Fig 5, S4 –S6 Tables). The median incidence was 84 per sample (range 1–481). The most common SVs were inversions (n = 256) and deletions (n = 232). Insertions were less commonly identified (n = 41) and were predominantly noncoding, with only one affecting a coding region of the gene *PSKH1*.

Recurrent structural variants were found in the *MTHFSD* gene (n = 4 samples), as well as *CDKN2A/B*, *DMD*, *DLG2, MTAP*, and *SETD2* (n = 3 samples each). Deletions were most common, although inversions, duplications, and translocations were identified in addition to deletions in some samples. In addition, several cancer-associated genes on chromosome 5 tended to be altered together or in similar ways, including *CBL*, *BCL9L*, *DDX6*, *DDX10*, *ATM*, *JUN*, *SKI*, *CAMTA1*, *PIK3CD*, *FANCA*, and *HYDIN*.

### Somatic copy number aberrations in canine oral OS

The most common copy number gains (log_2_ fold change ≥ 0.4) identified were in the tumor suppressor *ZFHX3* (5/8, 63%), the cytolytic granule protein gene *GNLY* (4/8, 50%), and the potassium voltage-gated channel gene *KCNQ3* (4/8, 50%) (S7 and S8 Tables, S3 Fig). Common copy number losses (log_2_ fold change ≤ −0.9) were seen s in *DLG2* (3/8, 38%), which has commonly been reported as deleted in OS [57]. Less common recurrent alterations included gains of the *RUNX2* oncogene and tumor suppressor *ARHGEF12* (3/8, 38%), and loss of *DMD* and the PI3K pathway tumor suppressor *PTEN* (2/8, 25%), all of which have been previously identified as altered in appendicular OS [9,53,57]. While copy number gains in *MYC* were present in fewer samples (1/8, 12%) than reported in canine appendicular OS (36%) [9], we lack statistical power to assess whether this is a true difference in affected driver genes or whether it is due to chance in a small cohort.

### Mutational signatures in canine oral OS

The trinucleotide context of SNVs was evaluated to identify mutational signatures defined by COSMIC v3. Signatures identified included: SBS1 (the “aging signature,” associated with spontaneous deamination of 5-methyl-cytosine), SBS8 (unknown etiology), SBS9 (possibly due to somatic hypermutation via polymerase eta in lymphoid cells), SBS17a (unknown etiology), SBS17b (associated in some human cases with fluorouracil chemotherapy and reactive oxygen species damage), SBS28 (unknown etiology) (Fig 4, S4 Fig) [32]. SBS1 made the highest contribution, while the lowest was made by SBS28. Many of these signatures have been previously reported in human or canine OS, with the exception of SBS28 [9,49,50].

### Pathways analysis of canine oral OS samples

We performed pathway analysis of 2785 genes with any recurrent coding mutation (altered in two or more samples). Of these, 2259 genes mapped to identifiers in the STRING Database. These genes were most enriched in immune-related pathways such as the KEGG Cytokine-cytokine receptor interaction (FDR = 1.7E-3) and Hippo signaling (FDR = 6.6E-3) pathways (S9 Table). The 183 genes with a coding SNV/INDEL in any sample (as too few genes had recurrent mutations to allow pathway analysis) mapped to 154 identifiers. These coding SNVs/INDELs were enriched in pathways including KEGG Central Carbon Metabolism in Cancer (FDR = 0.0046) and PI3K-Akt Signaling (FDR = 0.029), and the GO molecular function Protein tyrosine phosphatase activity (FDR = 0.022) (S10 Table). In contrast, the 1479 genes with recurrent noncoding mutations (UTR, intron,1025 identifiers) were most enriched in the “Nervous System” tissue compartment (FDR = 1.42E-28) as well as pathways including the Reactome “Neuronal system” (FDR = 4.01E-16) (S11 Table). Investigating this enrichment further, we noted that 4/8 samples had 3’UTR mutations in genes in the “Neurexins and Neuroligins” pathway (*NRXN1*, *NRXN3*, *DLGAP2*, and *EPB41L3*). Overall, 7/8 of our samples had some kind of alteration in this pathway (S11 Table).

Previous studies of canine appendicular OS have highlighted common mutations in the PI3K and MAPK pathways and genes involved in cell cycle and chromatin remodeling [9,53]. We investigated how many genes in each pathway were mutated in oral osteosarcoma by the different mutation types (S12 Table). The PI3K/AKT pathway was most commonly altered, with 6/8 samples having simple somatic mutations, 6/8 samples having structural variants, and 5/8 samples having somatic copy number aberrations. Chromatin Modification and Epigenetic Pathways were also commonly affected, 4/8 samples by simple somatic mutations, 6/8 samples by structural variants, and 5/8 samples by somatic copy number aberrations.

## Discussion

We sought to explore the genomic landscape of canine OS to determine whether genetic features of OS were conserved between appendicular and oral tumors. Overall, we found similarities between oral OS and reported genomic features of appendicular OS in dogs. Structural variants predominate in both tumor locations. *SETD2* and *DMD* were mutated in half of our samples, recapitulating the features of appendicular canine OS [9]. The p53 and PI3K pathways were altered in 38% of samples, and genes involved in chromatin regulation were altered in 50% of samples, frequencies similar to those previously reported in appendicular OS [9].

We identified several genomic features in this cohort to prioritize for further exploration in larger cohorts. The most common copy number gains were in *ZFHX3* and *KCNQ3*. *ZFHX3* is a transcription factor and negative regulator of c-Myb [58] that has been previously reported as gained in canine OS [9,59], and has been shown to be altered in a mutually exclusive manner with *MYC* in human cancers [60]. *KCNQ3* encodes a potassium voltage-gated channel which has been shown to increase MYC and Wnt pathway activity in gastroesophageal adenocarcinomas [61]. All samples in our study had copy number gains in either *ZFHX3*, *KCNQ3*, or *MYC*, while two samples had concurrent alterations in *ZFHX3* and *KCNQ3*, suggesting that alterations in these genes may have a similar or complimentary functional role in oral OS tumors.

Another interesting feature of our dataset was the recurrent alteration of the neurexin and neuroligin pathway, which includes the *DLG2* gene commonly altered in appendicular OS. While genes in this pathway are mainly known for their role in synapses, they have also been shown to play an important role in the vascular system [62–64]. In cancer, they promote colorectal cancer progression through the APC/β-catenin pathway [65], are overexpressed in non-small-cell lung cancer [66], are associated with poor prognosis in Ewing’s sarcoma [67], and have been reported to facilitate bone cancer pain [68]. Collectively, our findings highlight neurexins and neuroligins as candidates for further study in oral OS.

The primary limitation of this exploratory study is the small sample size. Larger studies will be essential to generate a more comprehensive understanding of genetic alterations in oral OS and to compare and contrast genomic alterations between appendicular and oral OS in dogs and humans. An additional limitation of this study was the low tumor fraction of some of our samples. In our study, two samples (Axial-OS-01 and Axial-OS-03) had ichorCNA-estimated tumor fractions below 10%, which likely hindered our ability to identify somatic alterations in these samples. Due to the small sample size and lack of comprehensive clinical metadata, we were also unable to explore the potential effects of tumor subtype, grade, and stage on the tumor genome.

## Conclusions

Our canine oral osteosarcoma dataset is defined by high structural complexity and few recurrent point mutations and demonstrates broad genetic concordance between oral and appendicular OS. We highlight copy number gains in *ZFHX3* and *KCNQ3* and alteration of the neurexin and neuroligin pathway for further exploration. Prospective studies with larger sample sizes will be necessary to further characterize the genetic landscape of oral OS.

## Supporting information

S1 Table Clinical metadata and tumor fraction. Clinical metadata including breed, age, sex, tumor site, tumor size, ichorCNA tumor fraction, and treatment information.
(XLSX)

S2 Table Sequencing information. Sequencing metrics from platform for each axial osteosarcoma sample.
(XLSX)

S3 Table Simple somatic mutations. All simple somatic mutations that passed filters.
(XLSX)

S4 Table Structural variants. Structural variants called by Manta that passed filters, annotated by Snpeff.
(XLSX)

S5 Table Chromosome-level structural variants. Structural variants that are classified “chromosomal” and the genes within those regions in each sample.
(XLSX)

S6 Table Coding structural variants. **A.** Translocations, **B.** Deletions, **C.** Duplications, **D.** Insertions, **E.** Inversions. Samples with no coding variants are omitted.
(XLSX)

S7 Table Somatic copy number aberrations. Copy number variants and overlapping genes within the variant regions called in each axial osteosarcoma sample.
(XLSX)

S8 Table Coding somatic copy number aberrations. Somatic copy number aberrations and overlapping genes filtered to variants that overlap exon regions of genes.
(XLSX)

S9 Table Pathway enrichment of all mutation types. Results of STRING Database pathway enrichment for all genes with mutations in two or more samples.
(XLSX)

S10 Table Pathway enrichment of coding mutations. Results of STRING Database pathway enrichment for all genes with coding mutations.
(XLSX)

S11 Table Pathway enrichment of noncoding simple somatic mutations. Results of STRING Database pathway enrichment of mutations non-coding simple somatic mutations seen in two or more samples.
(XLSX)

S12 Table Somatic mutations in commonly altered pathways. Somatic mutations in commonly altered pathways in OS.
(XLSX)

S1 Fig Tumor histology. Images of hematoxylin and eosin-stained histology slides from samples **A.** Axial-OS-02, **B.** Axial-OS-04, and **C.** Axial-OS-05.
(PDF)

S2 Fig Rainfall plots. Rainfall plots for every axial osteosarcoma sample with density plots with distance between mutations in log_10_ scales.
(PDF)

S3 Fig Copy number segmentation plots. Denoised copy number segmentation plots with the copy number segments represented in blue and orange. Black line represents the denoised median.
(PDF)

S4 Fig Mutational signature plots. Mutational signature composition of each sample.
(PDF)
