# Supplementary figures and images for: Characterization of the genomic landscape of canine oral osteosarcoma reveals similarities with appendicular osteosarcoma
Source: PLoS One. 2025 Jun 10;20(6):e0325181. doi: 10.1371/journal.pone.0325181 (PMC12151373; doi:10.1371/journal.pone.0325181)

# Supplemental Figure 1

**A** Axial-OS-02

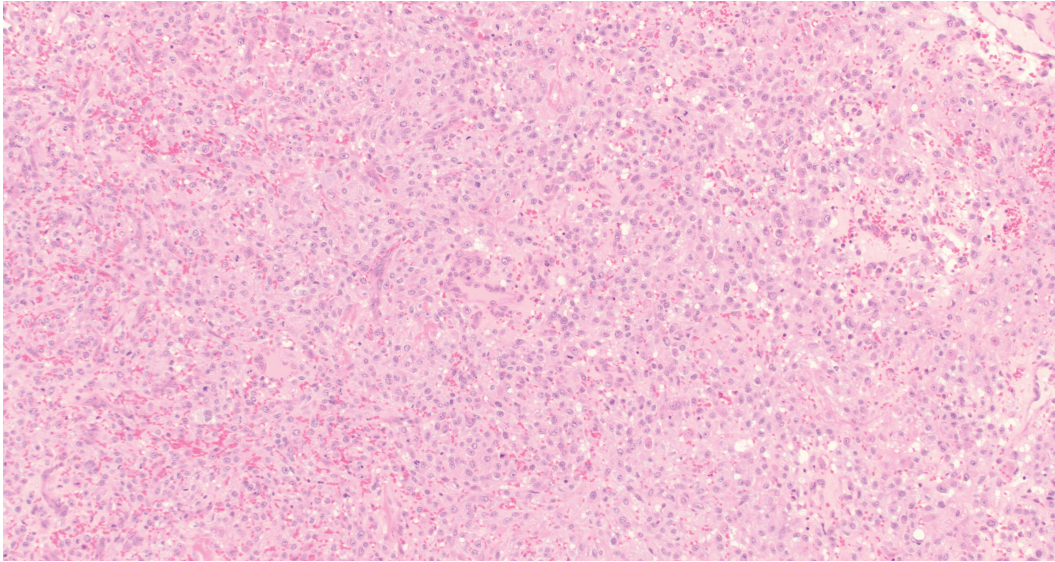

**B** Axial-OS-04

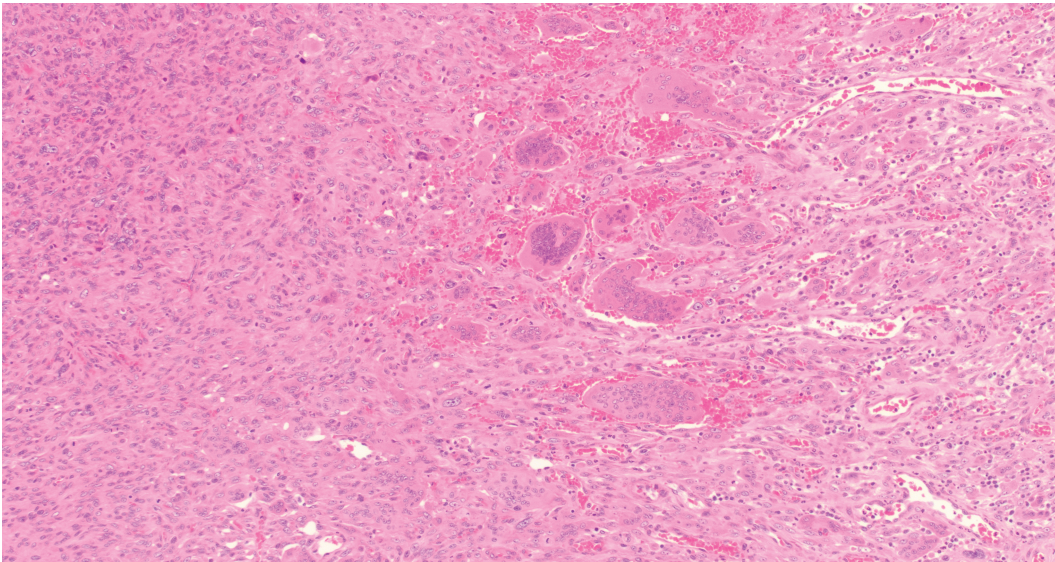

**C** Axial-OS-05

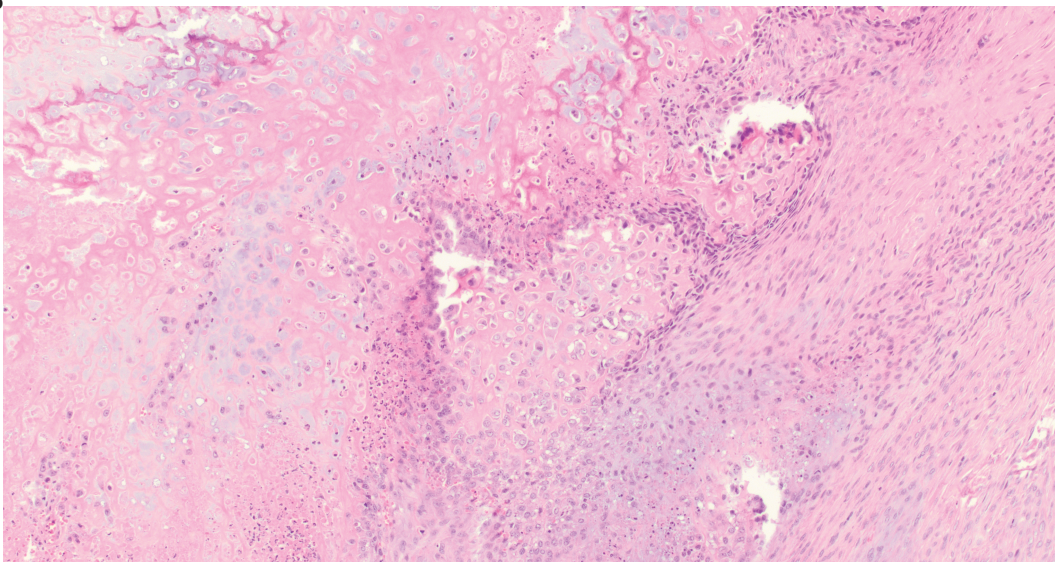

Supplement: S1 Fig — Images of hematoxylin and eosin-stained histology slides from samples A. Axial-OS-02, B. Axial-OS-04, and C. Axial-OS-05. (PDF) [file pone.0325181.s013.pdf]

# Supplemental Figure 3

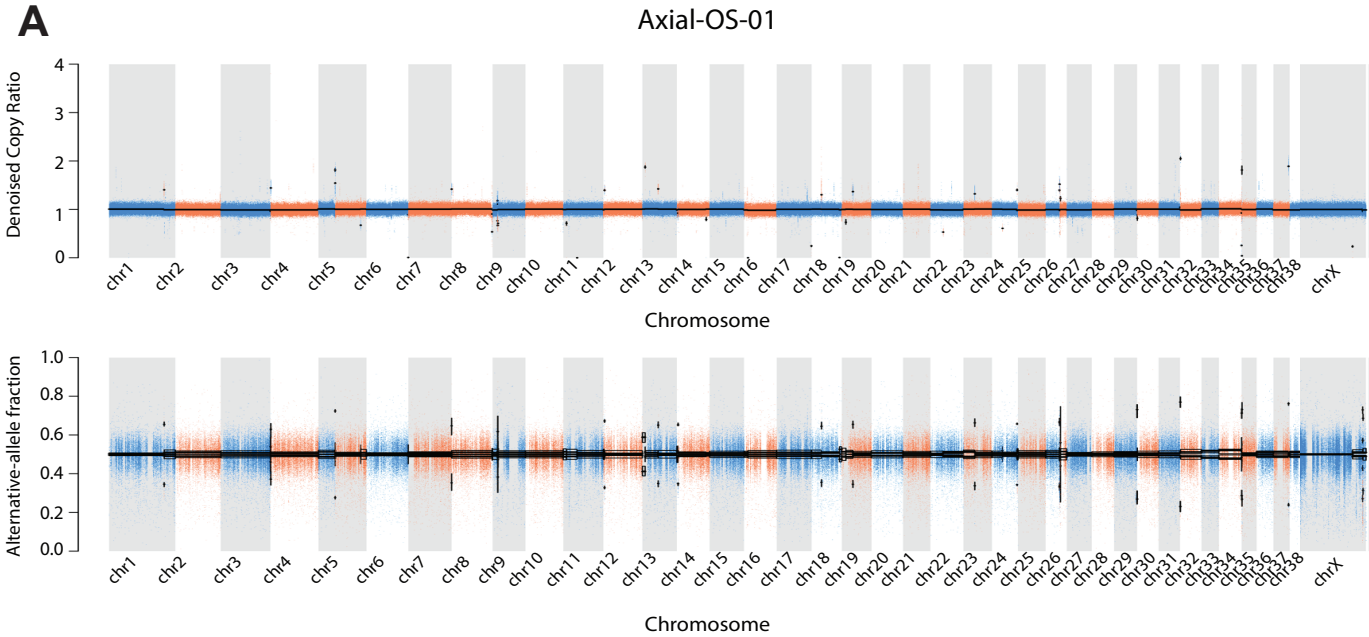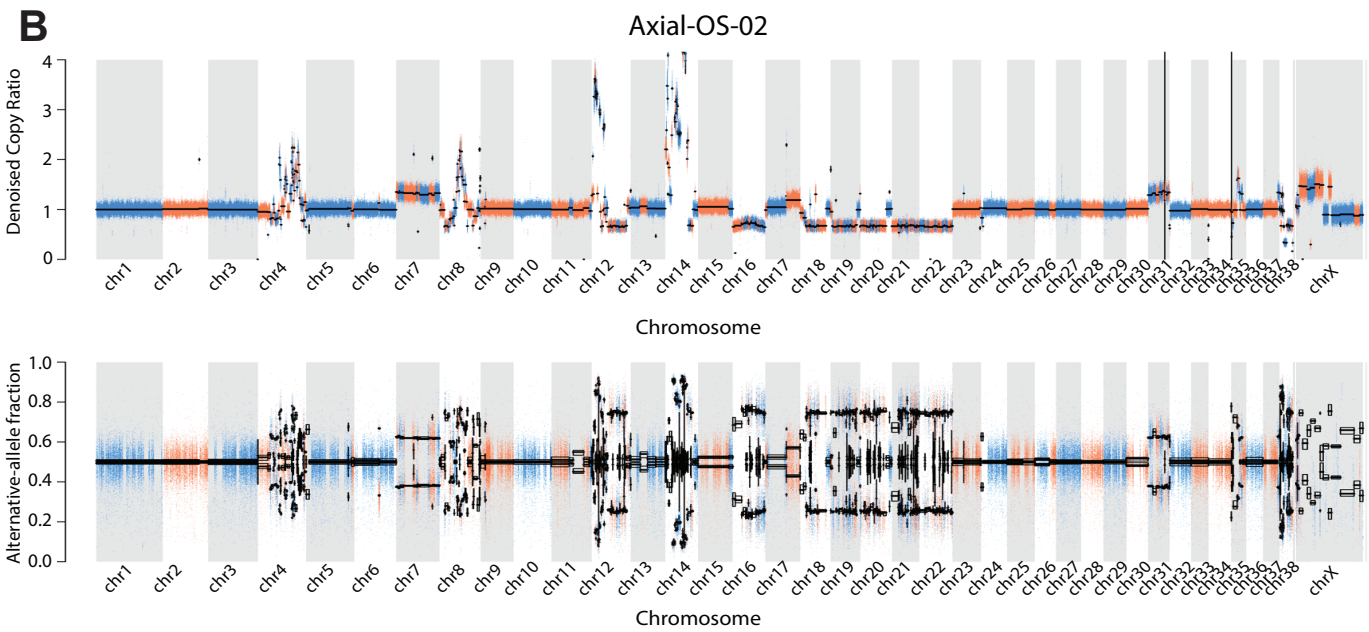

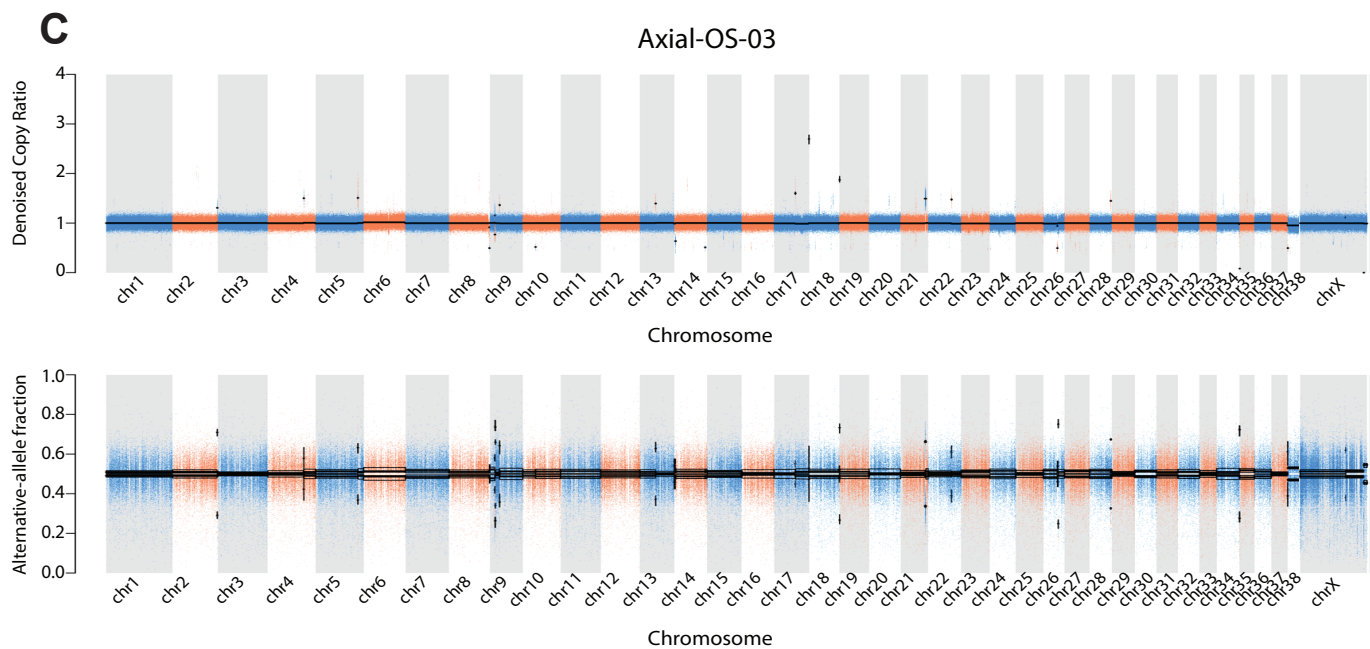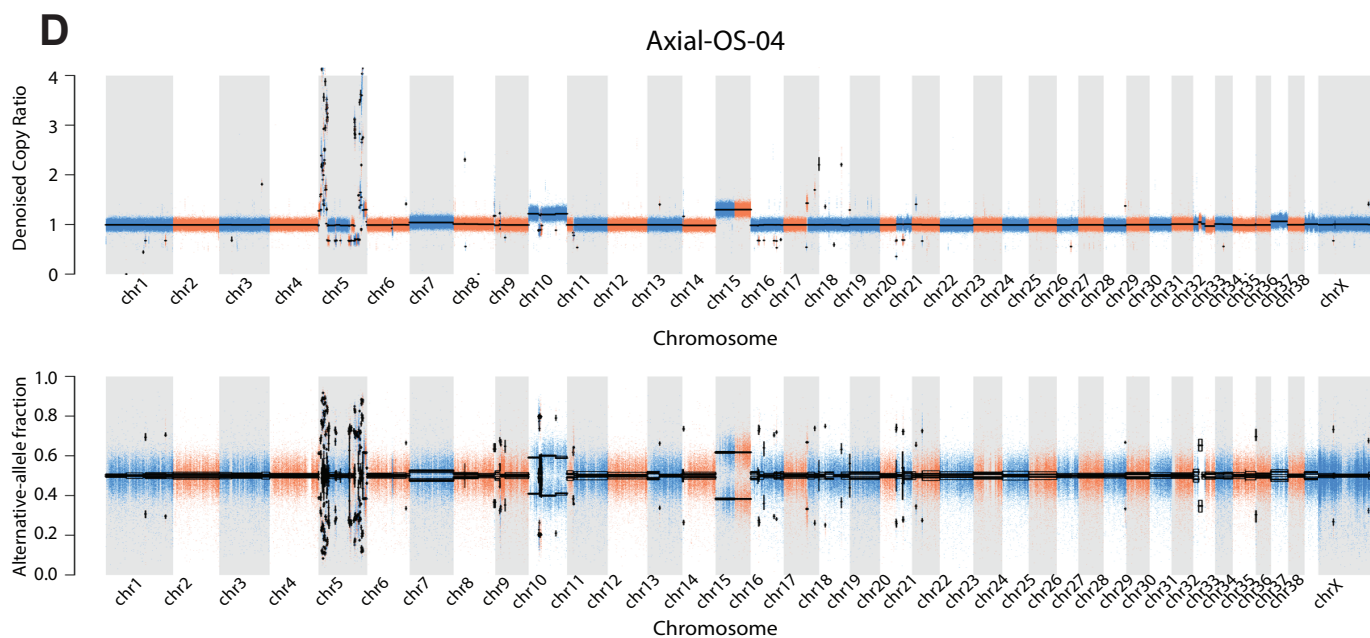

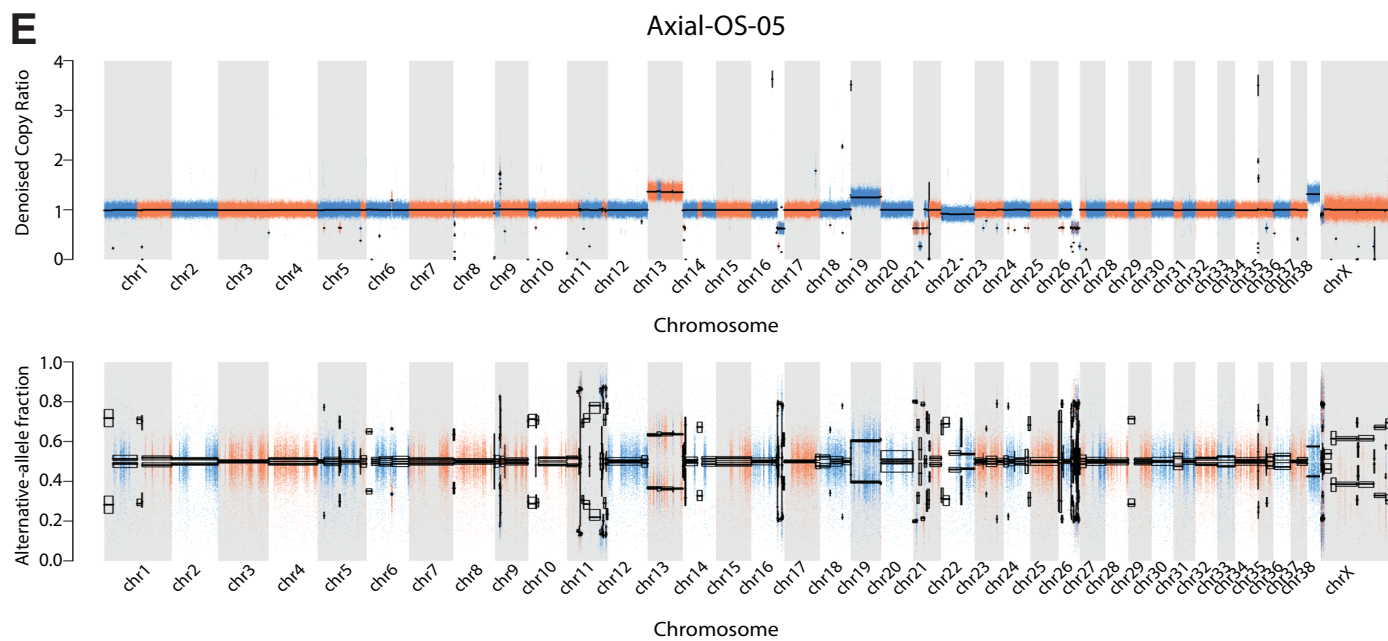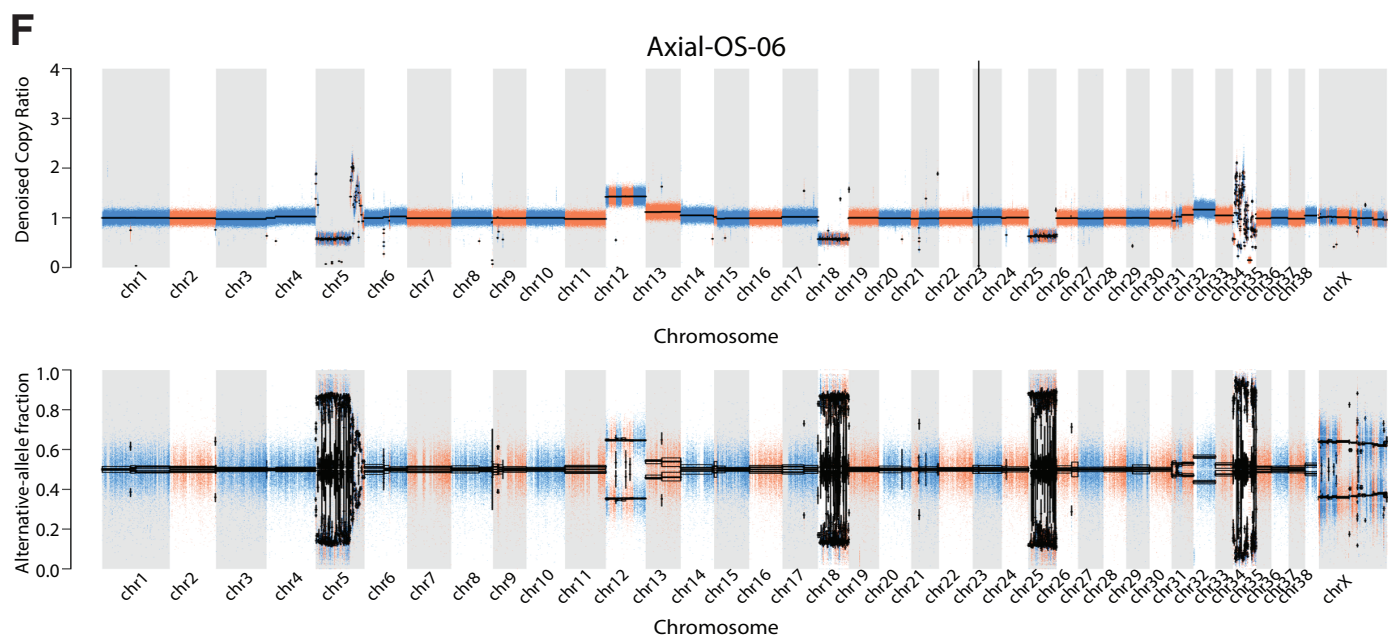

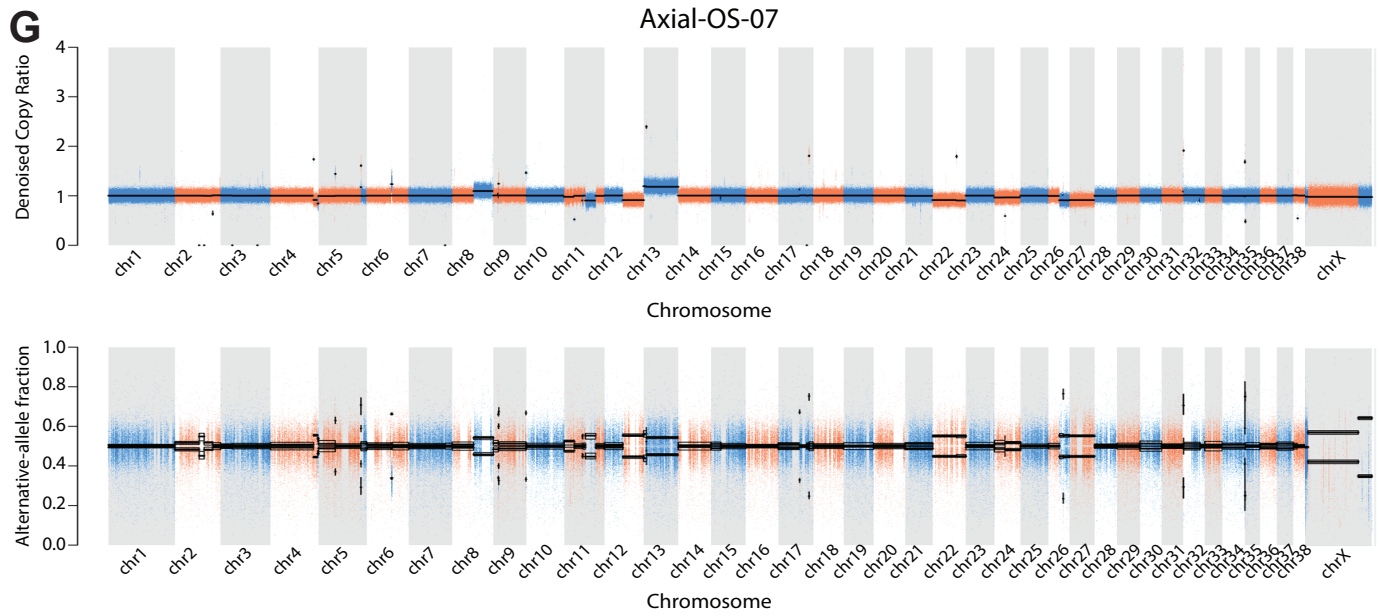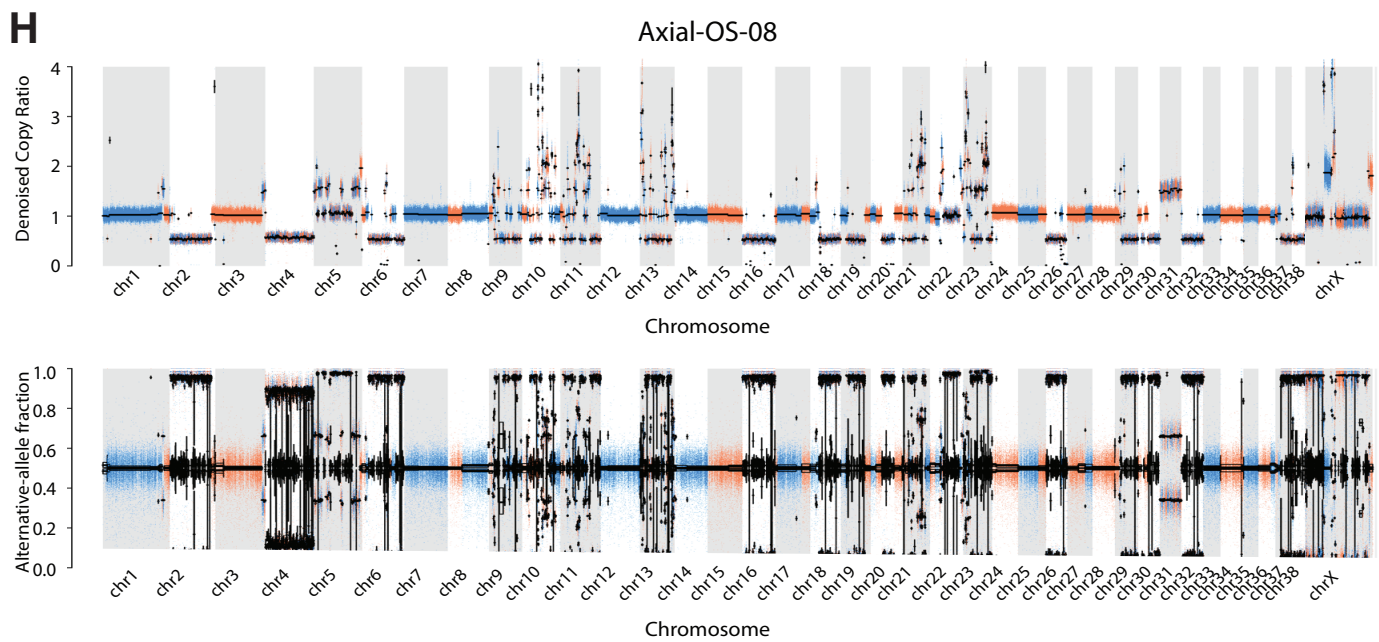

Supplement: S3 Fig — Denoised copy number segmentation plots with the copy number segments represented in blue and orange. Black line represents the denoised median. (PDF) [file pone.0325181.s015.pdf]
