# Supplementary material for: Characterization of the genomic landscape of canine oral osteosarcoma reveals similarities with appendicular osteosarcoma
Source: PLoS One. 2025 Jun 10;20(6):e0325181. doi: 10.1371/journal.pone.0325181 (PMC12151373; doi:10.1371/journal.pone.0325181)
Supplement: S2 Fig — Rainfall plots for every axial osteosarcoma sample with density plots with distance between mutations in log10 scales. (PDF) [file pone.0325181.s014.pdf]

Supplemental Figure 2

A

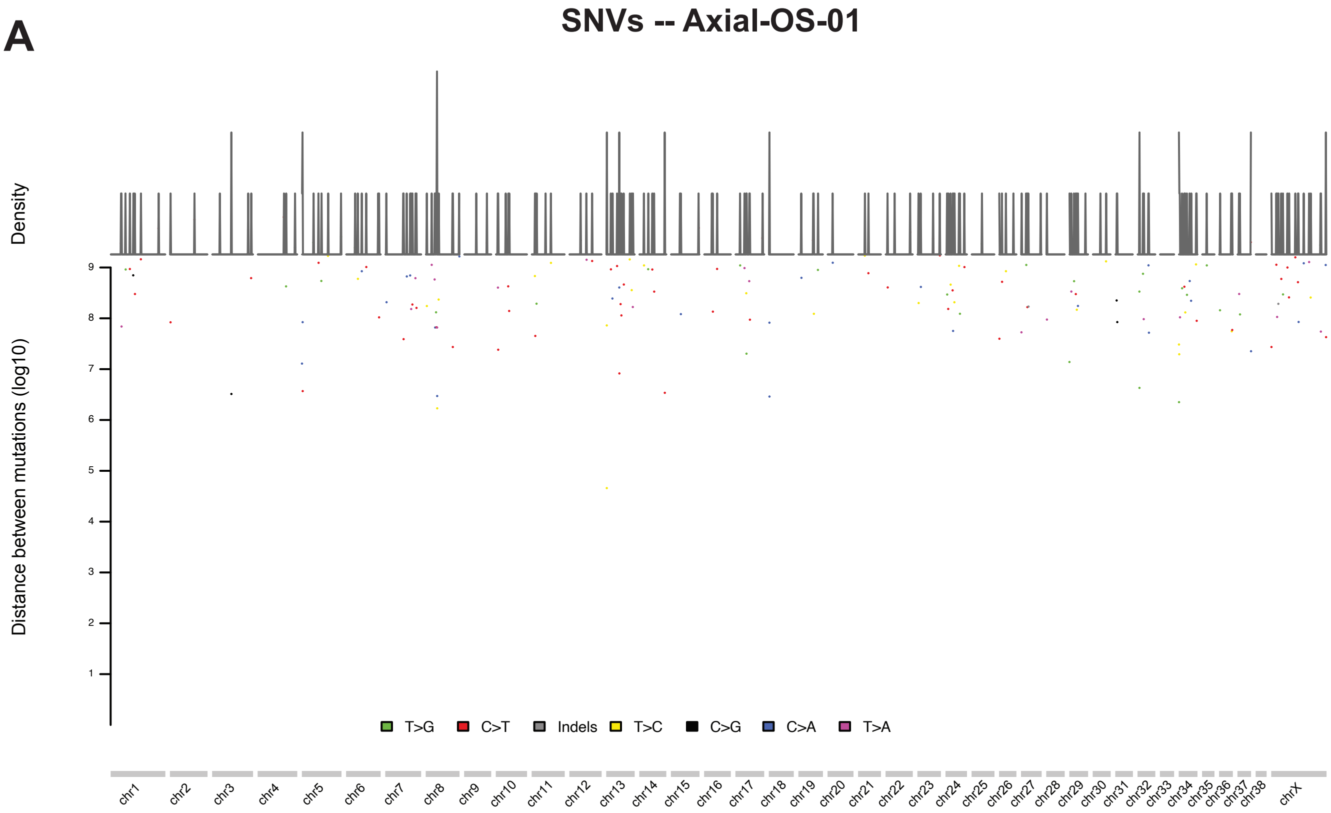

B

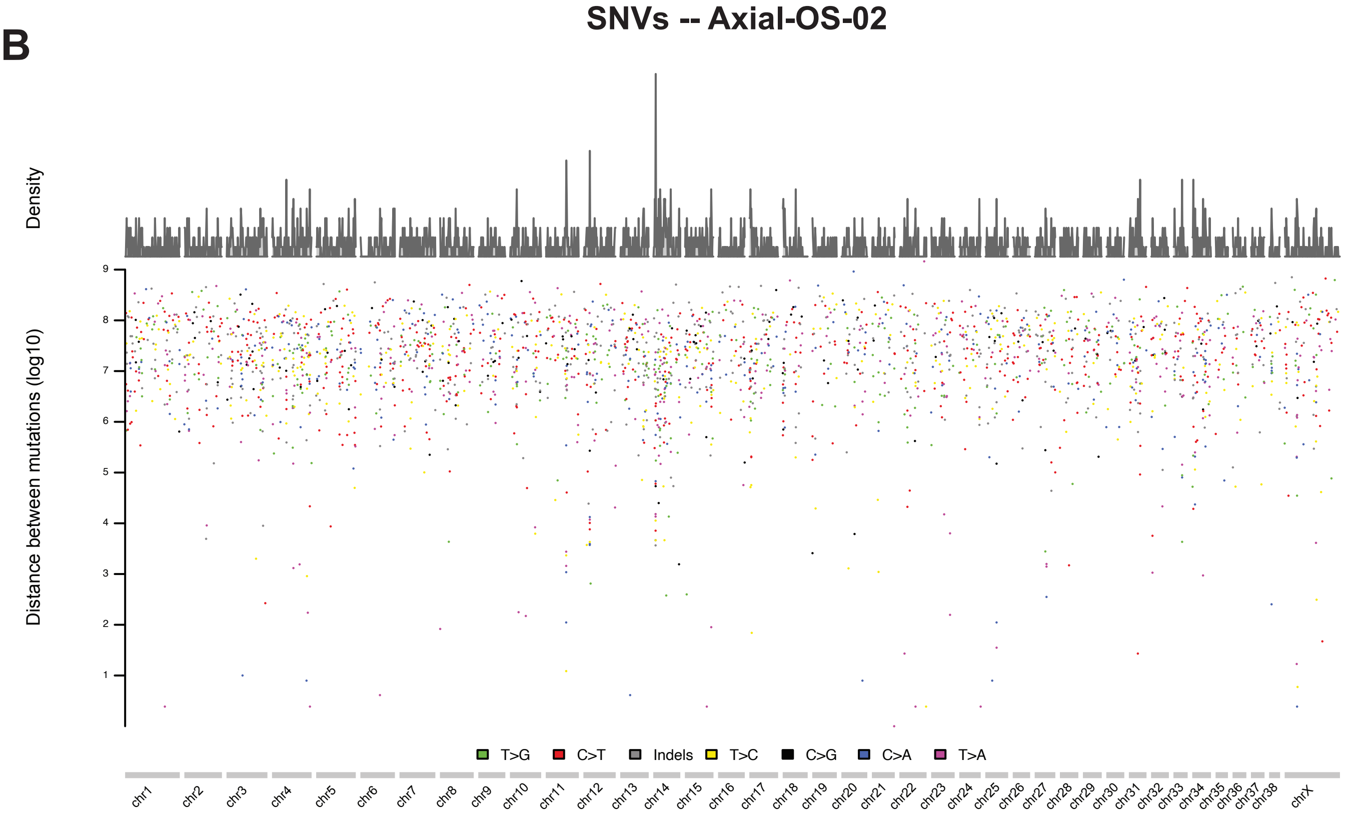

C

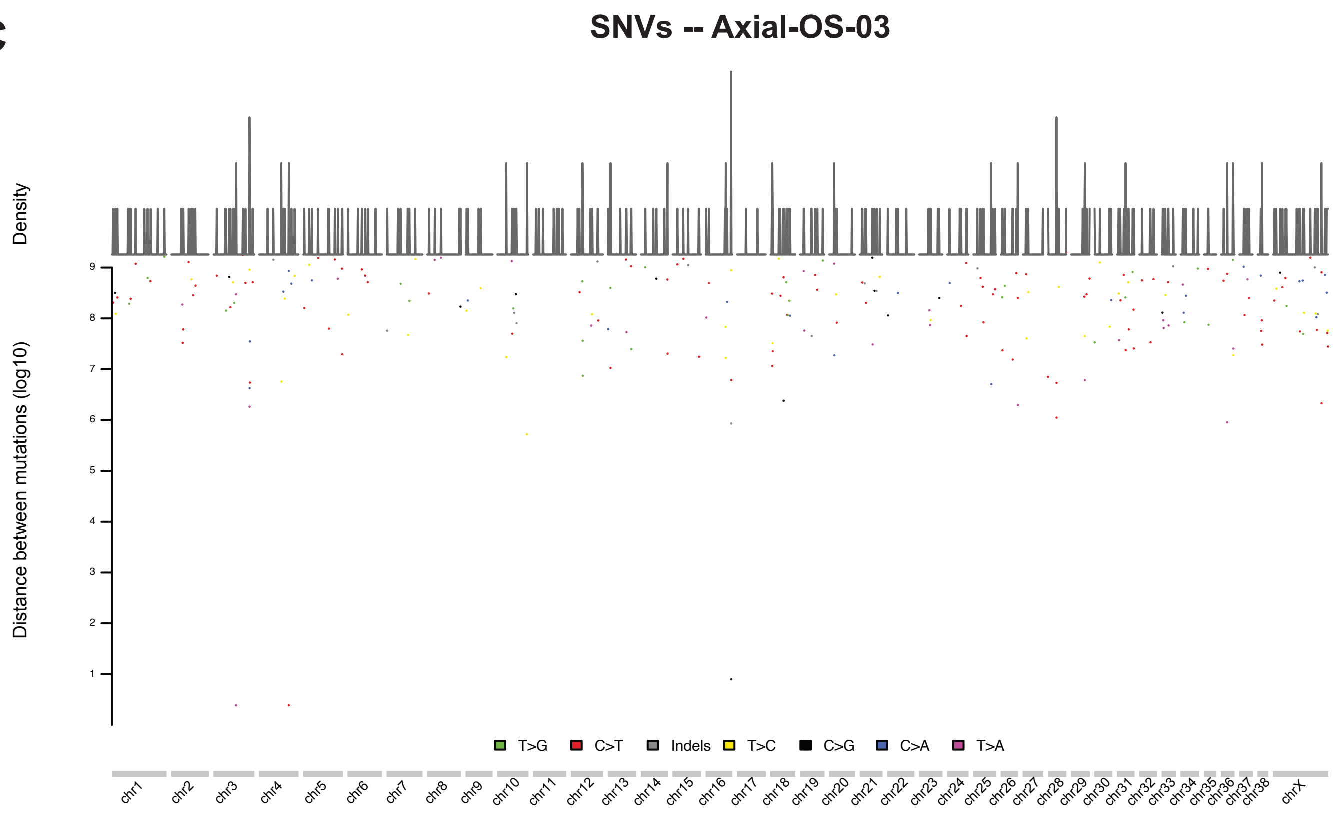
SNVs -- Axial-OS-03
Density
Distance between mutations (log10)
T>G
C>T
Indels
T>C
C>G
C>A
T>A
chr1
chr2
chr3
chr4
chr5
chr6
chr7
chr8
chr9
chr10
chr11
chr12
chr13
chr14
chr15
chr16
chr17
chr18
chr19
chr20
chr21
chr22
chr23
chr24
chr25
chr26
chr27
chr28
chr29
chr30
chr31
chr32
chr33
chr34
chr35
chr36
chr37
chr38
chrX

D

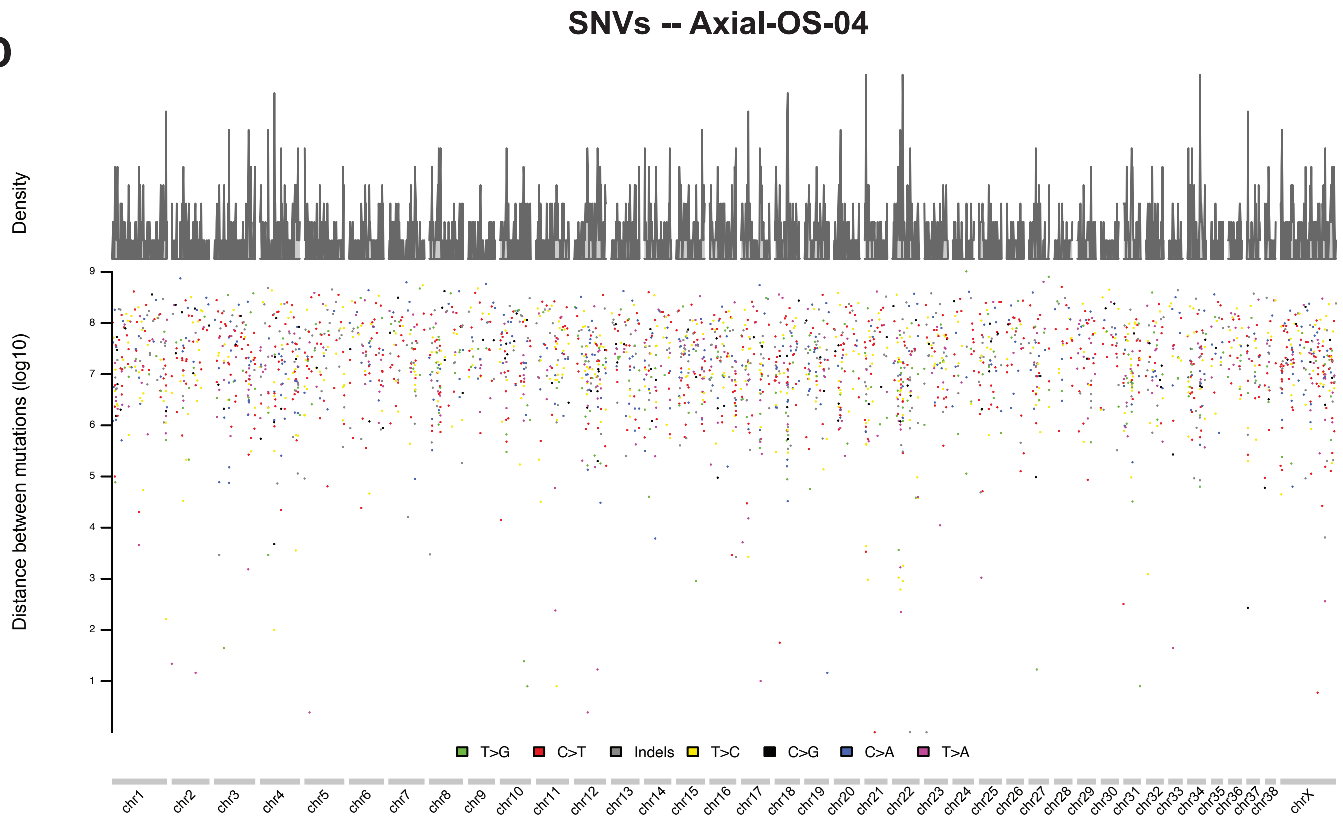
SNVs -- Axial-OS-04
Density
Distance between mutations (log10)
T>G
C>T
Indels
T>C
C>G
C>A
T>A
chr1
chr2
chr3
chr4
chr5
chr6
chr7
chr8
chr9
chr10
chr11
chr12
chr13
chr14
chr15
chr16
chr17
chr18
chr19
chr20
chr21
chr22
chr23
chr24
chr25
chr26
chr27
chr28
chr29
chr30
chr31
chr32
chr33
chr34
chr35
chr36
chr37
chr38
chrX

E

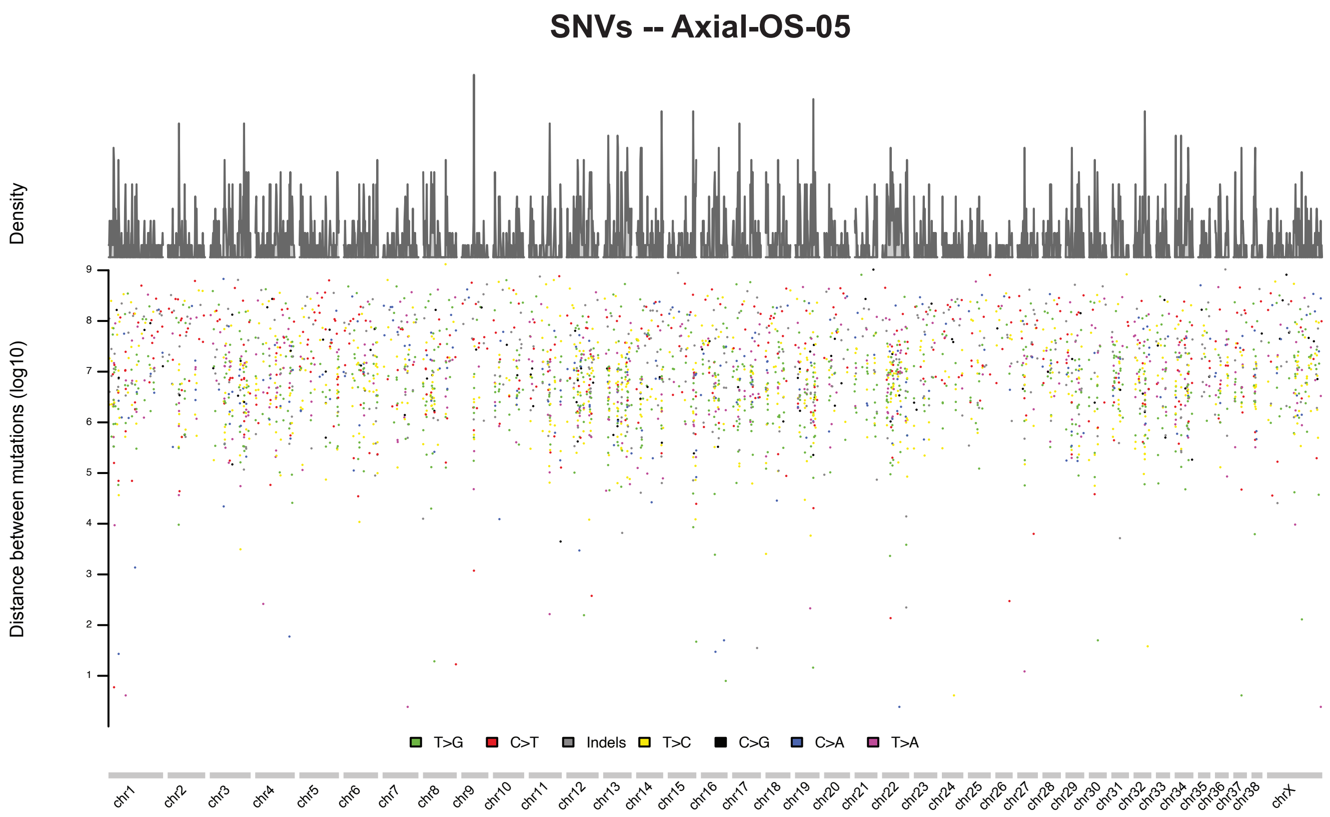
SNVs -- Axial-OS-05
Density
Distance between mutations (log10)
T>G C>T Indels T>C C>G C>A T>A
chr1 chr2 chr3 chr4 chr5 chr6 chr7 chr8 chr9 chr10 chr11 chr12 chr13 chr14 chr15 chr16 chr17 chr18 chr19 chr20 chr21 chr22 chr23 chr24 chr25 chr26 chr27 chr28 chr29 chr30 chr31 chr32 chr33 chr34 chr35 chr36 chr37 chr38 chrX

F

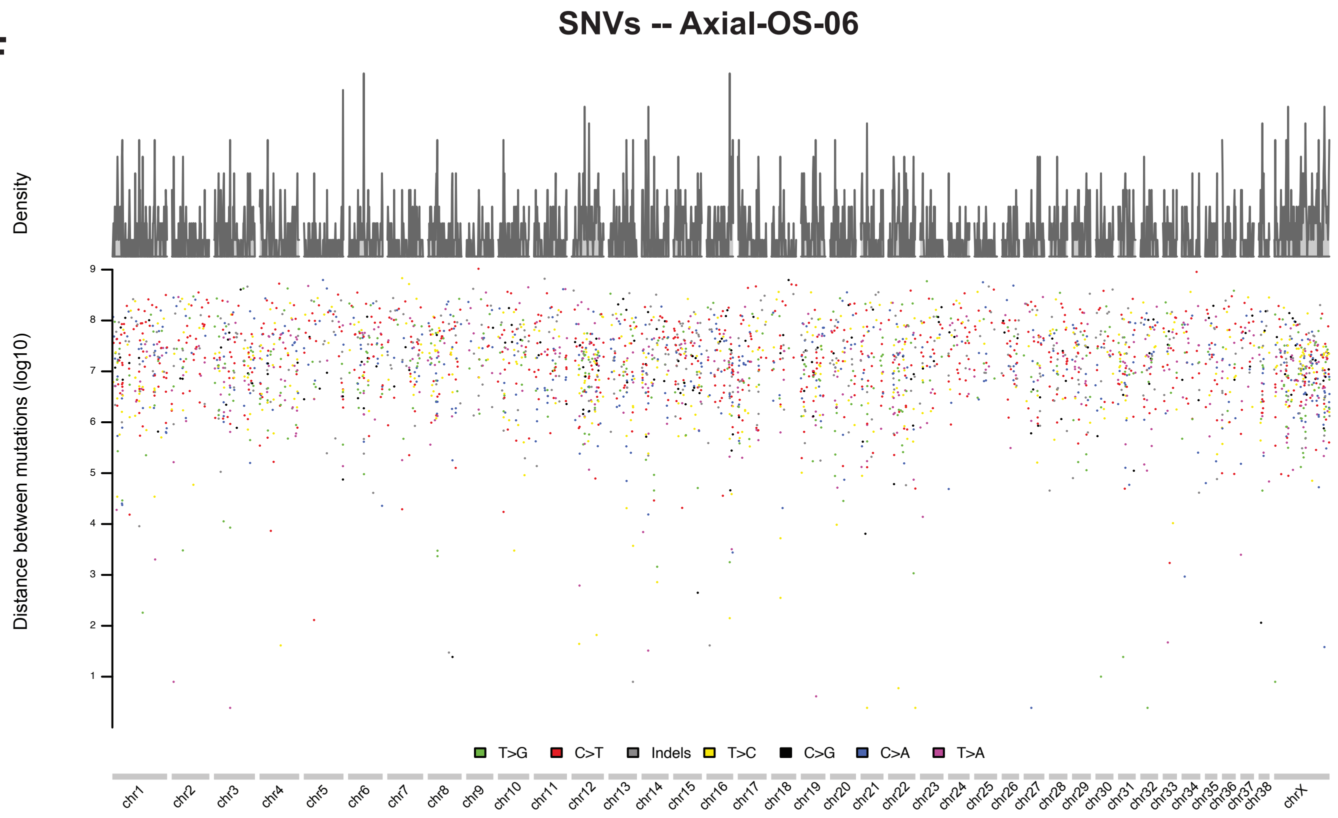
SNVs -- Axial-OS-06
Density
Distance between mutations (log10)
T>G C>T Indels T>C C>G C>A T>A
chr1 chr2 chr3 chr4 chr5 chr6 chr7 chr8 chr9 chr10 chr11 chr12 chr13 chr14 chr15 chr16 chr17 chr18 chr19 chr20 chr21 chr22 chr23 chr24 chr25 chr26 chr27 chr28 chr29 chr30 chr31 chr32 chr33 chr34 chr35 chr36 chr37 chr38 chrX

G

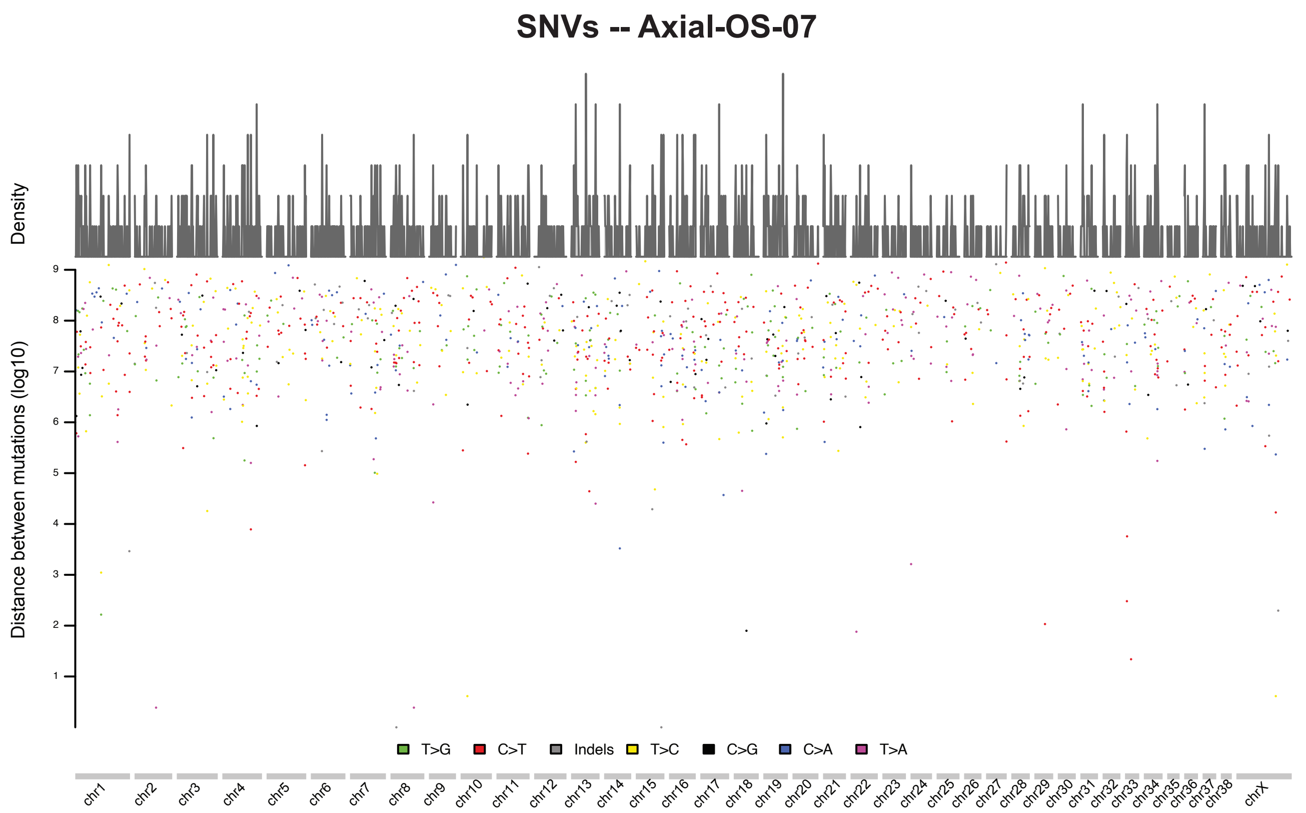
SNVs -- Axial-OS-07
Density
Distance between mutations (log10)
1
2
3
4
5
6
7
8
9
T>G
C>T
Indels
T>C
C>G
C>A
T>A
chr1 chr2 chr3 chr4 chr5 chr6 chr7 chr8 chr9 chr10 chr11 chr12 chr13 chr14 chr15 chr16 chr17 chr18 chr19 chr20 chr21 chr22 chr23 chr24 chr25 chr26 chr27 chr28 chr29 chr30 chr31 chr32 chr33 chr34 chr35 chr36 chr37 chr38 chrX

H

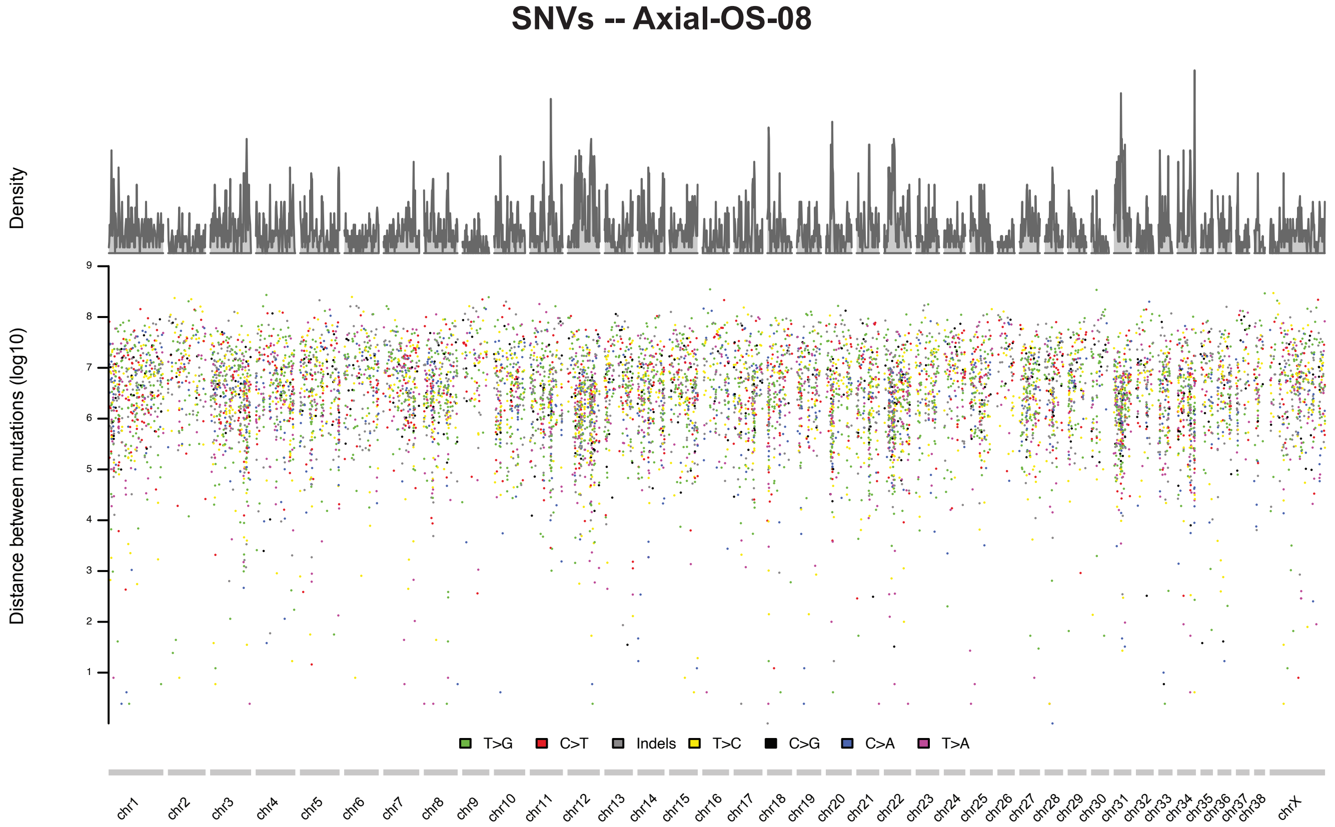
SNVs -- Axial-OS-08
Density
Distance between mutations (log10)
1
2
3
4
5
6
7
8
9
T>G
C>T
Indels
T>C
C>G
C>A
T>A
chr1 chr2 chr3 chr4 chr5 chr6 chr7 chr8 chr9 chr10 chr11 chr12 chr13 chr14 chr15 chr16 chr17 chr18 chr19 chr20 chr21 chr22 chr23 chr24 chr25 chr26 chr27 chr28 chr29 chr30 chr31 chr32 chr33 chr34 chr35 chr36 chr37 chr38 chrX
