# Supplementary material for: Characterization of the genomic landscape of canine oral osteosarcoma reveals similarities with appendicular osteosarcoma
Source: PLoS One. 2025 Jun 10;20(6):e0325181. doi: 10.1371/journal.pone.0325181 (PMC12151373; doi:10.1371/journal.pone.0325181)
Supplement: S4 Fig — Mutational signature composition of each sample. (PDF) [file pone.0325181.s016.pdf]

Supplemental Figure 4

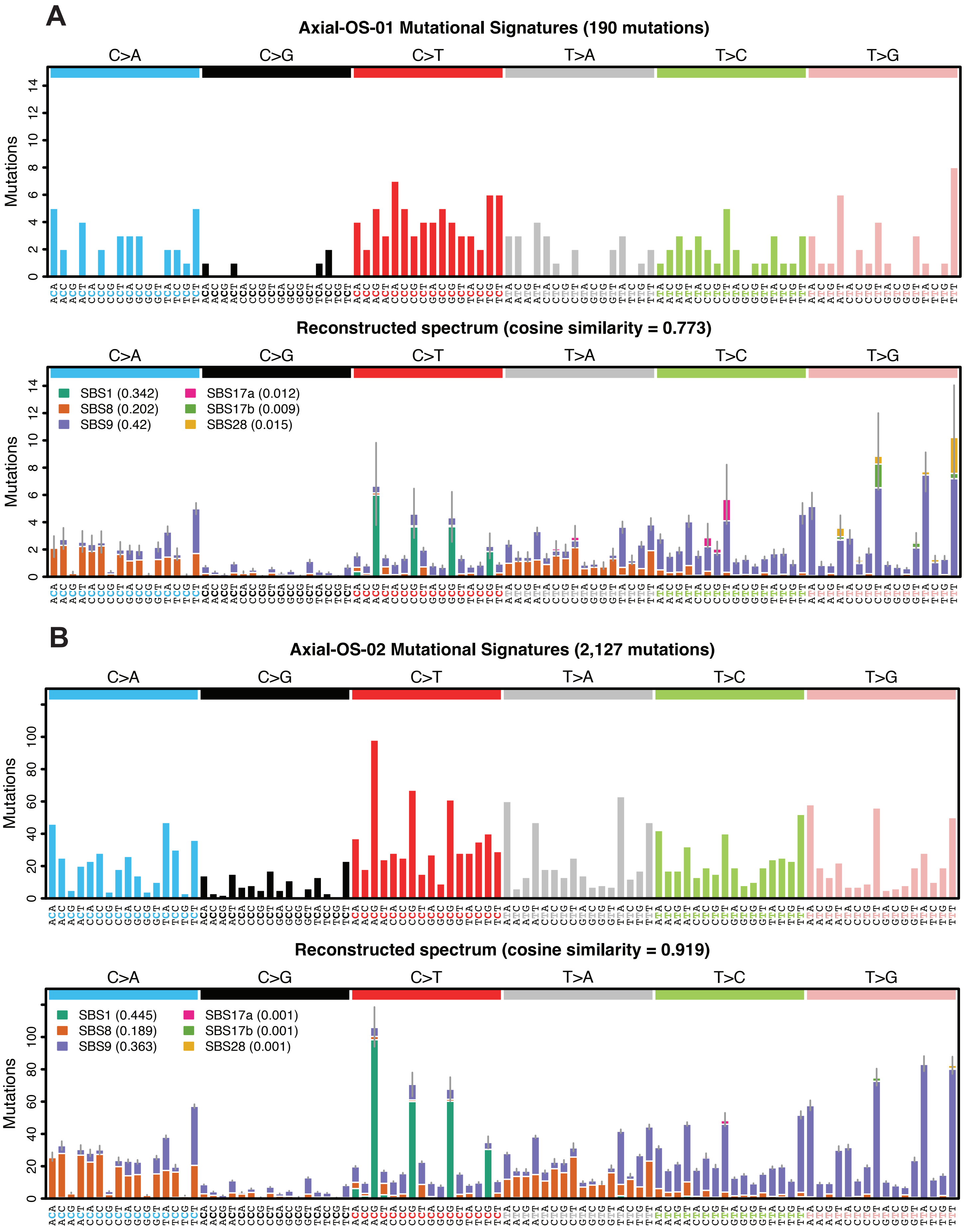

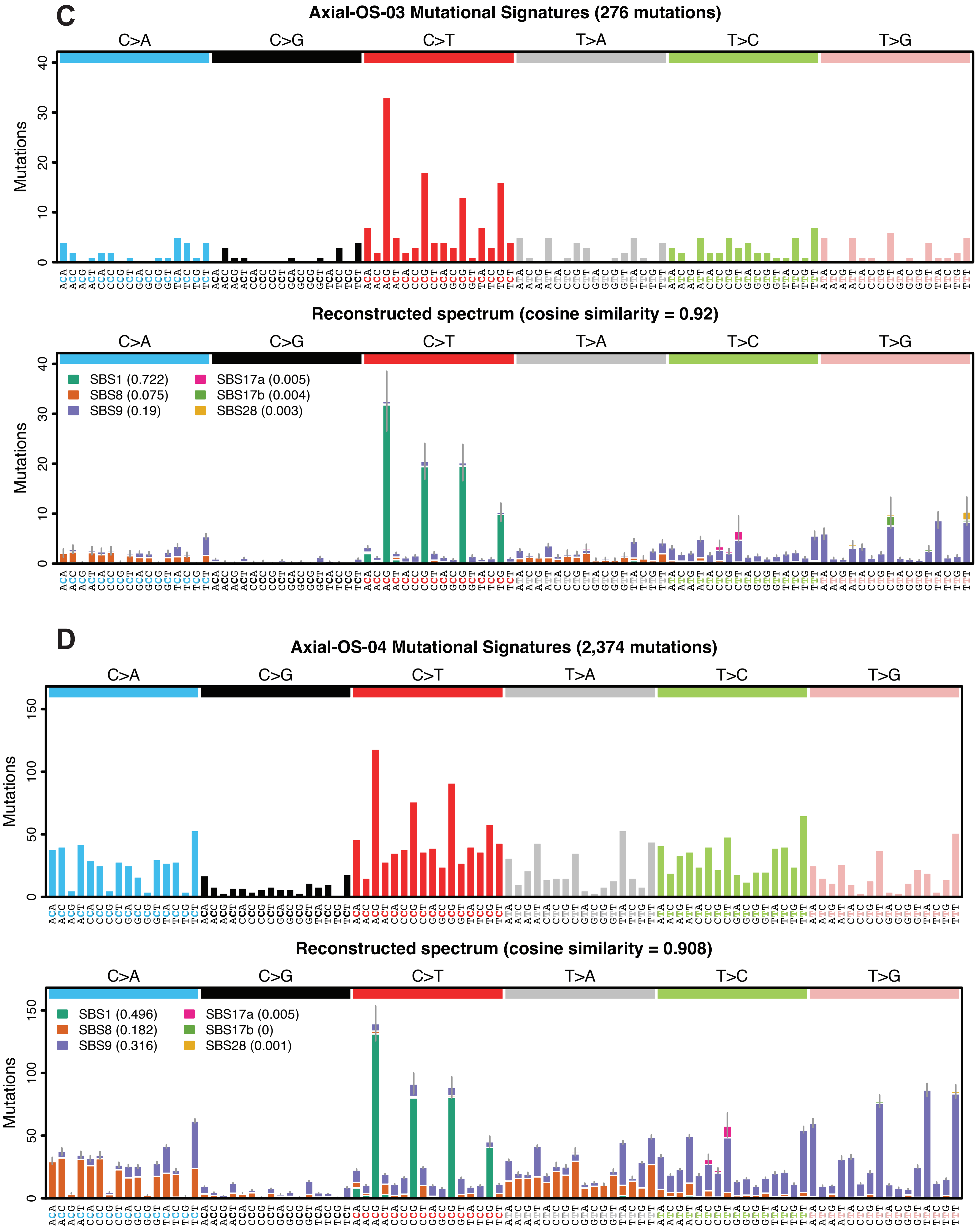
C
Axial-OS-03 Mutational Signatures (276 mutations)
C>A
C>G
C>T
T>A
T>C
T>G
Mutations
Reconstructed spectrum (cosine similarity = 0.92)
SBS1 (0.722)
SBS8 (0.075)
SBS9 (0.19)
SBS17a (0.005)
SBS17b (0.004)
SBS28 (0.003)
D
Axial-OS-04 Mutational Signatures (2,374 mutations)
Reconstructed spectrum (cosine similarity = 0.908)
SBS1 (0.496)
SBS8 (0.182)
SBS9 (0.316)
SBS17a (0.005)
SBS17b (0)
SBS28 (0.001)

E

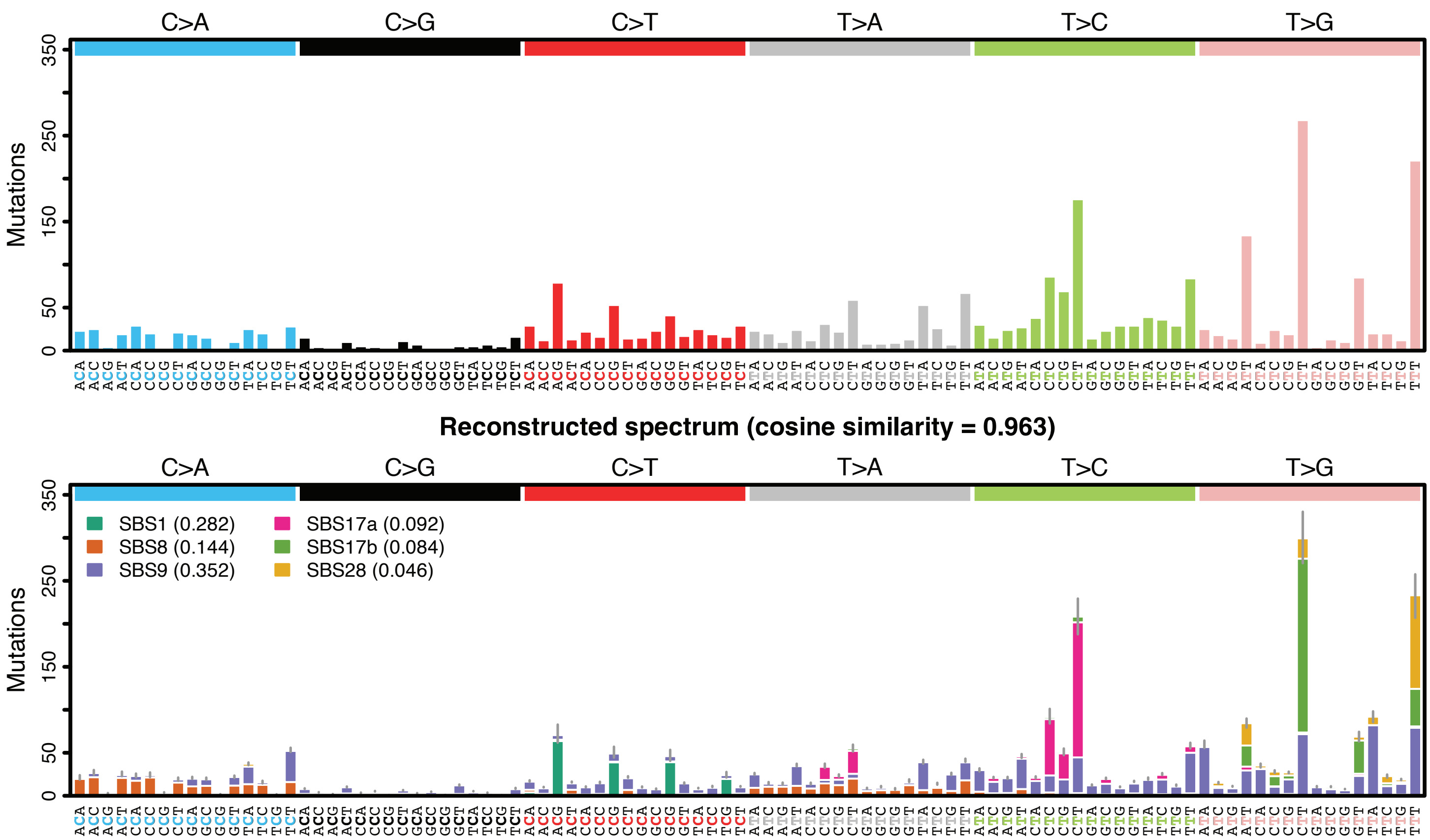

Axial-OS-05 Mutational Signatures (2,826 mutations)
C>A
C>G
C>T
T>A
T>C
T>G
Mutations
Reconstructed spectrum (cosine similarity = 0.963)
SBS1 (0.282)
SBS8 (0.144)
SBS9 (0.352)
SBS17a (0.092)
SBS17b (0.084)
SBS28 (0.046)

F

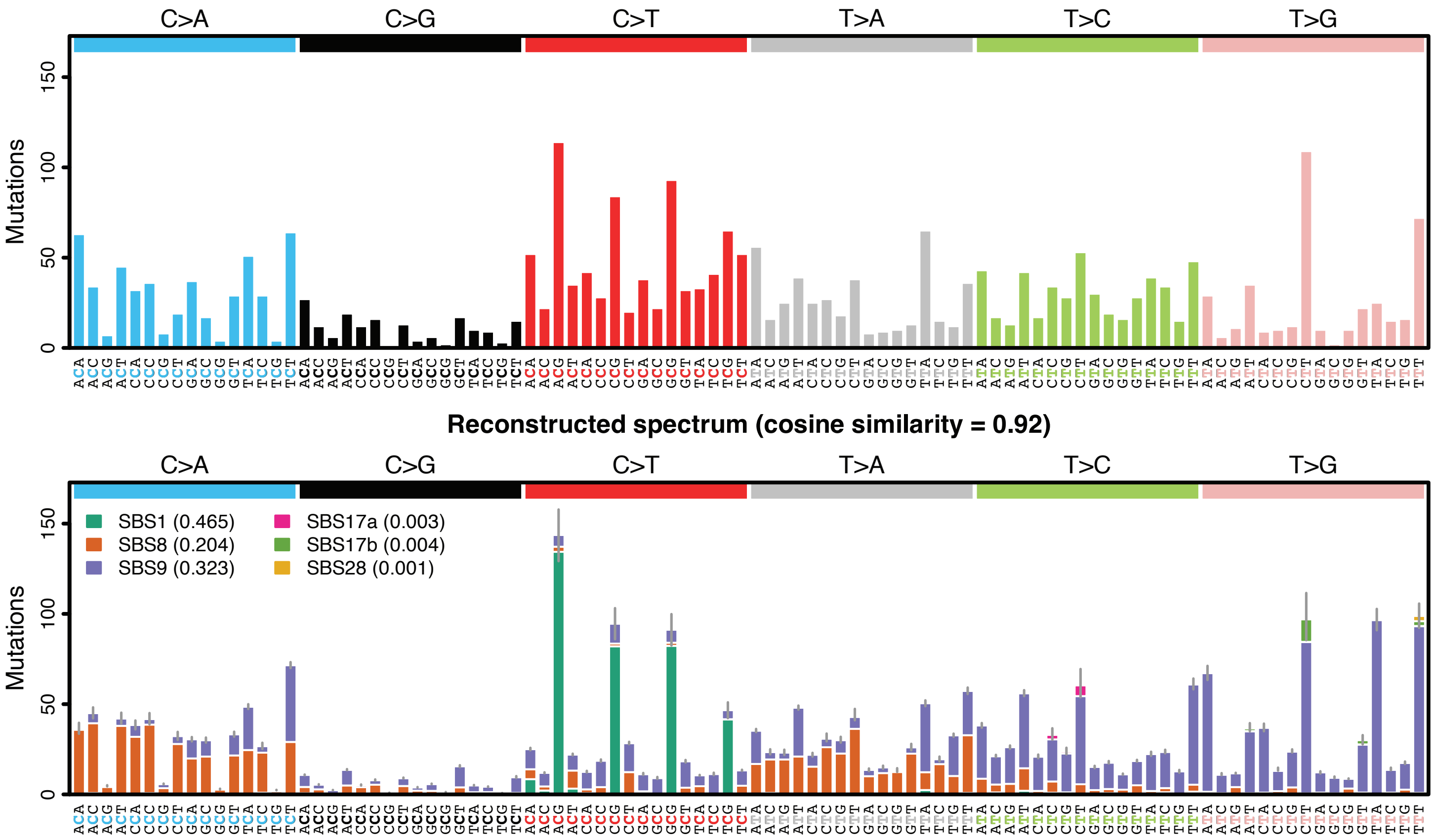

Axial-OS-06 Mutational Signatures (2,705 mutations)
C>A
C>G
C>T
T>A
T>C
T>G
Mutations
Reconstructed spectrum (cosine similarity = 0.92)
SBS1 (0.465)
SBS8 (0.204)
SBS9 (0.323)
SBS17a (0.003)
SBS17b (0.004)
SBS28 (0.001)

G

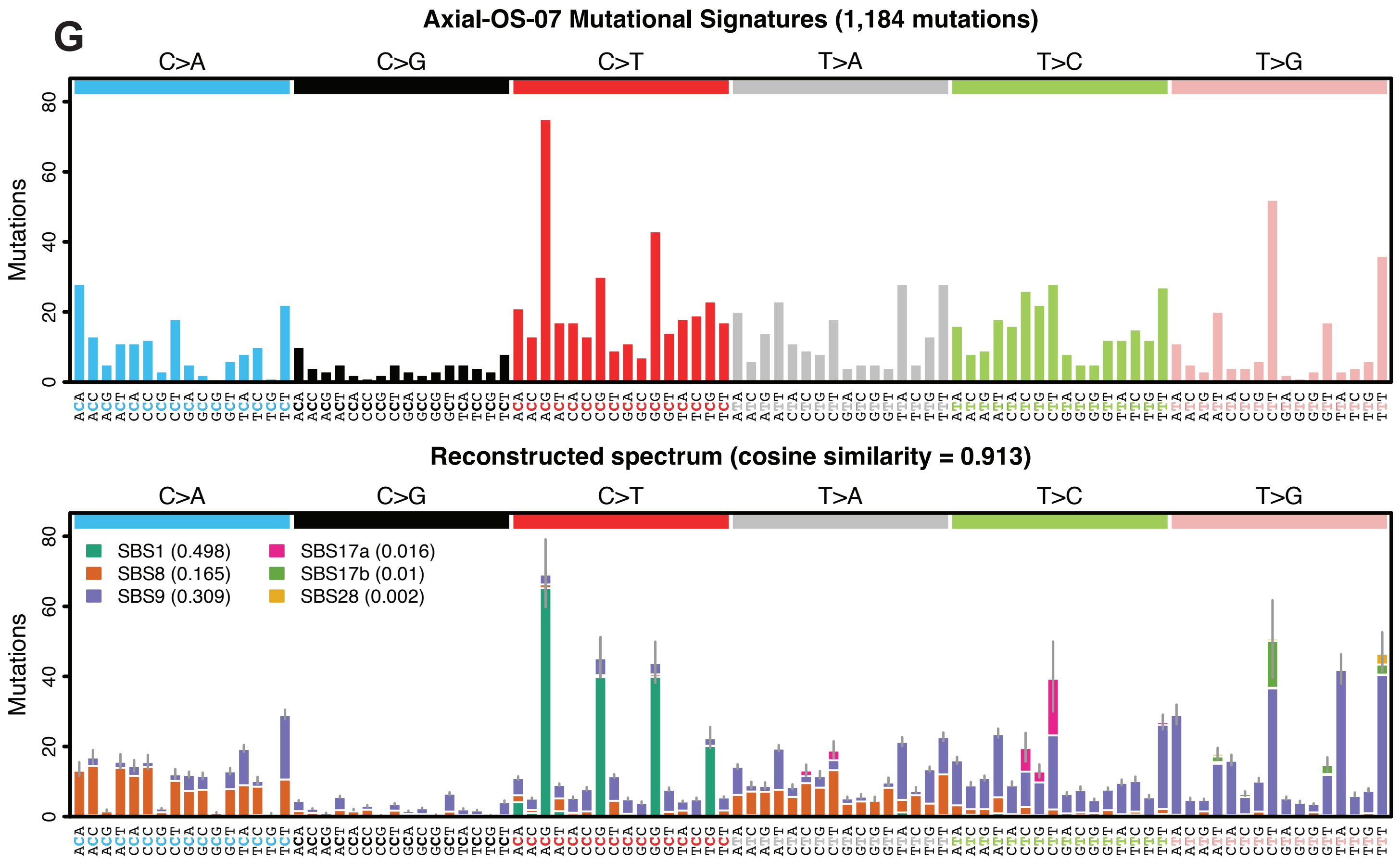

Axial-OS-07 Mutational Signatures (1,184 mutations)
C>A
C>G
C>T
T>A
T>C
T>G
Mutations
Reconstructed spectrum (cosine similarity = 0.913)
SBS1 (0.498)
SBS8 (0.165)
SBS9 (0.309)
SBS17a (0.016)
SBS17b (0.01)
SBS28 (0.002)

H

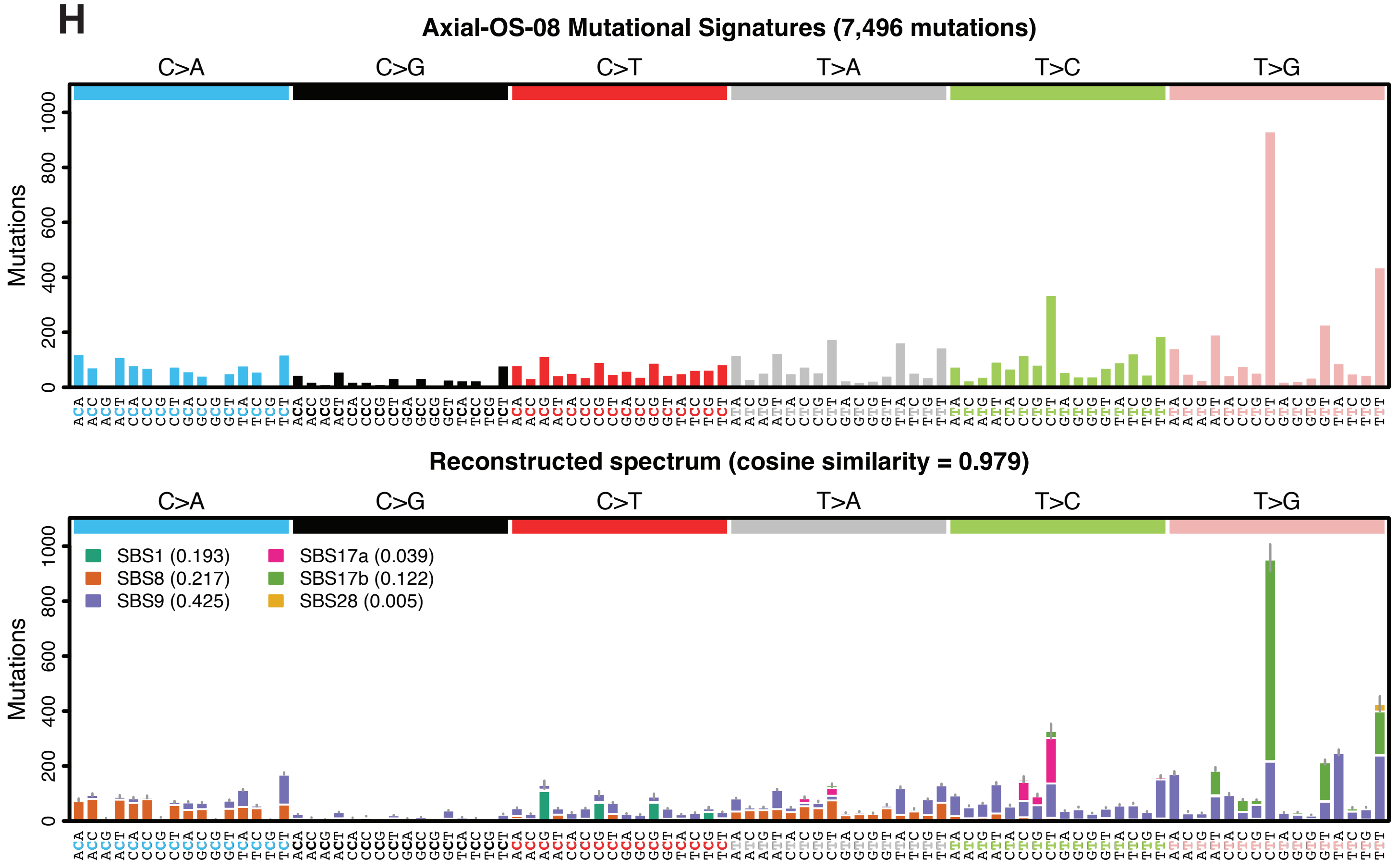

Axial-OS-08 Mutational Signatures (7,496 mutations)
C>A
C>G
C>T
T>A
T>C
T>G
Mutations
Reconstructed spectrum (cosine similarity = 0.979)
SBS1 (0.193)
SBS8 (0.217)
SBS9 (0.425)
SBS17a (0.039)
SBS17b (0.122)
SBS28 (0.005)
